# Supplementary figures and images for: Integrating precision medicine in the study and clinical treatment of a severely mentally ill person
Source: PeerJ. 2013 Oct 3;1:e177. doi: 10.7717/peerj.177 (PMC3792182; doi:10.7717/peerj.177)

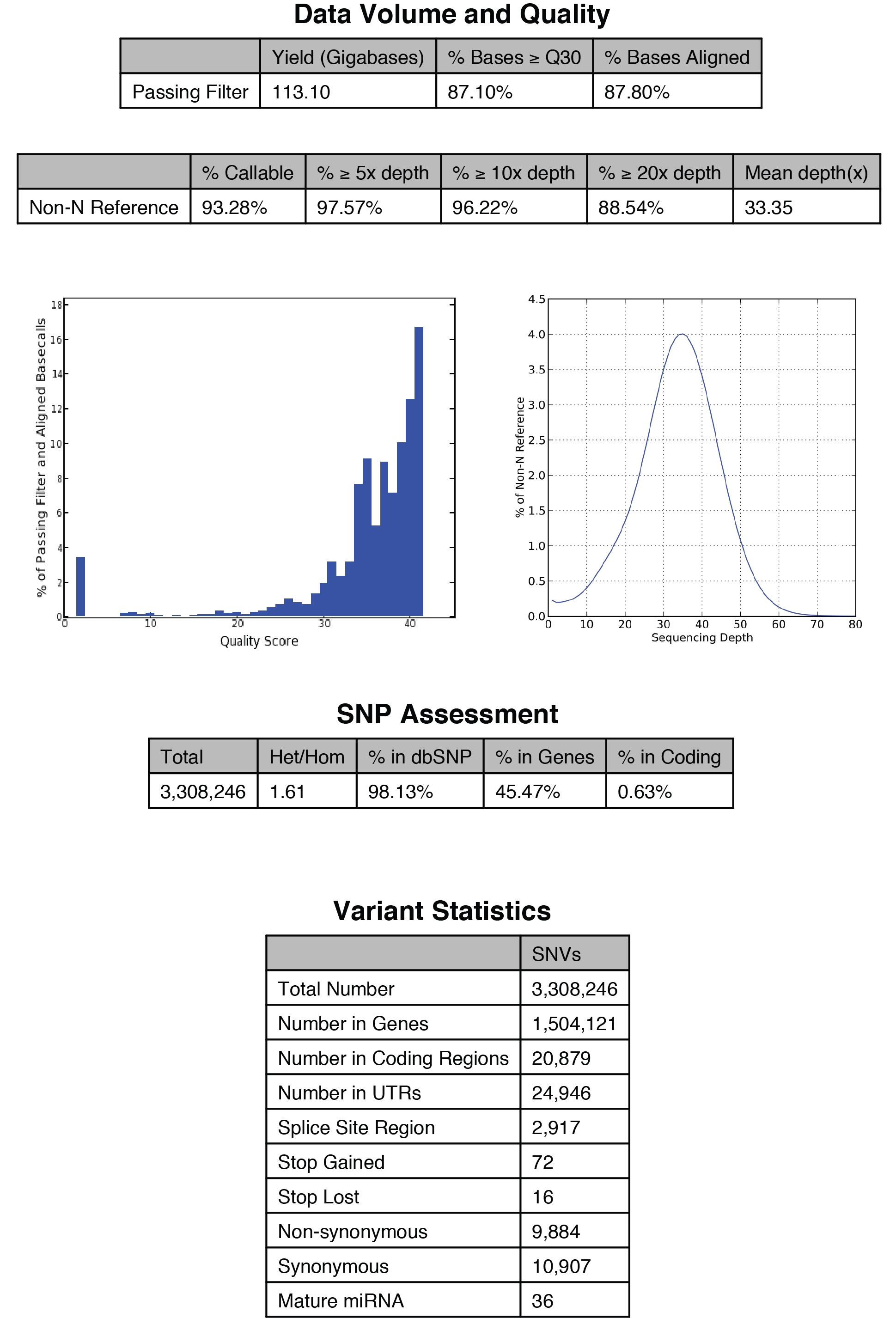

Supplement: Figure S1 — WGS was performed using the Illumina CLIA WGS pipeline. We report the volume of data, the quality of the data as well as whole genome SNP characteristics and more general characteristics of SNVs reported by the Illumina CLIA WGS pipeline, including: the total number of SNVs, the total number of SNVs that are within genes, coding regions, UTRs, splice site regions as well as the number of SNVs that were stop gained, stop lost, non-synonymous, synonymous and mature mRNA. [file peerj-01-177-s001.png]

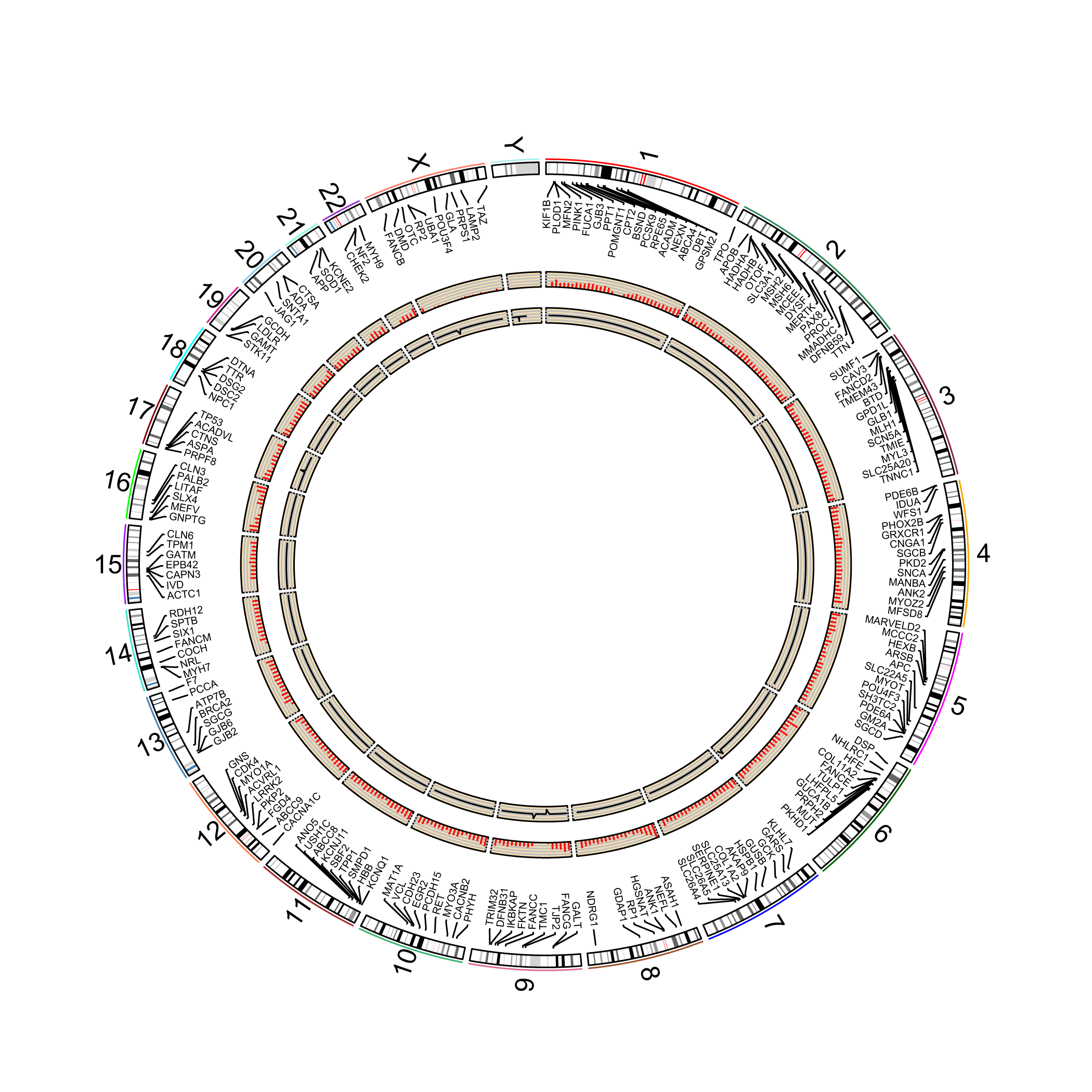

Supplement: Figure S2 — We show here a summary of the genomic coordinates corresponding to the 344 genes that were clinically evaluated by the Illumina CLIA WGS pipeline, the frequency of IGN validated SNVs across the genome (plotted in red) and a summary of highly confident copy number variants (CNVs) that were simultaneously detected by the Estimation by Read Depth with SNVs (ERDS) and Copy Number Analysis Method (CNAM) detection methods (plotted in black). Duplications and deletions are depicted as elevations and declinations, respectively. [file peerj-01-177-s003.png]

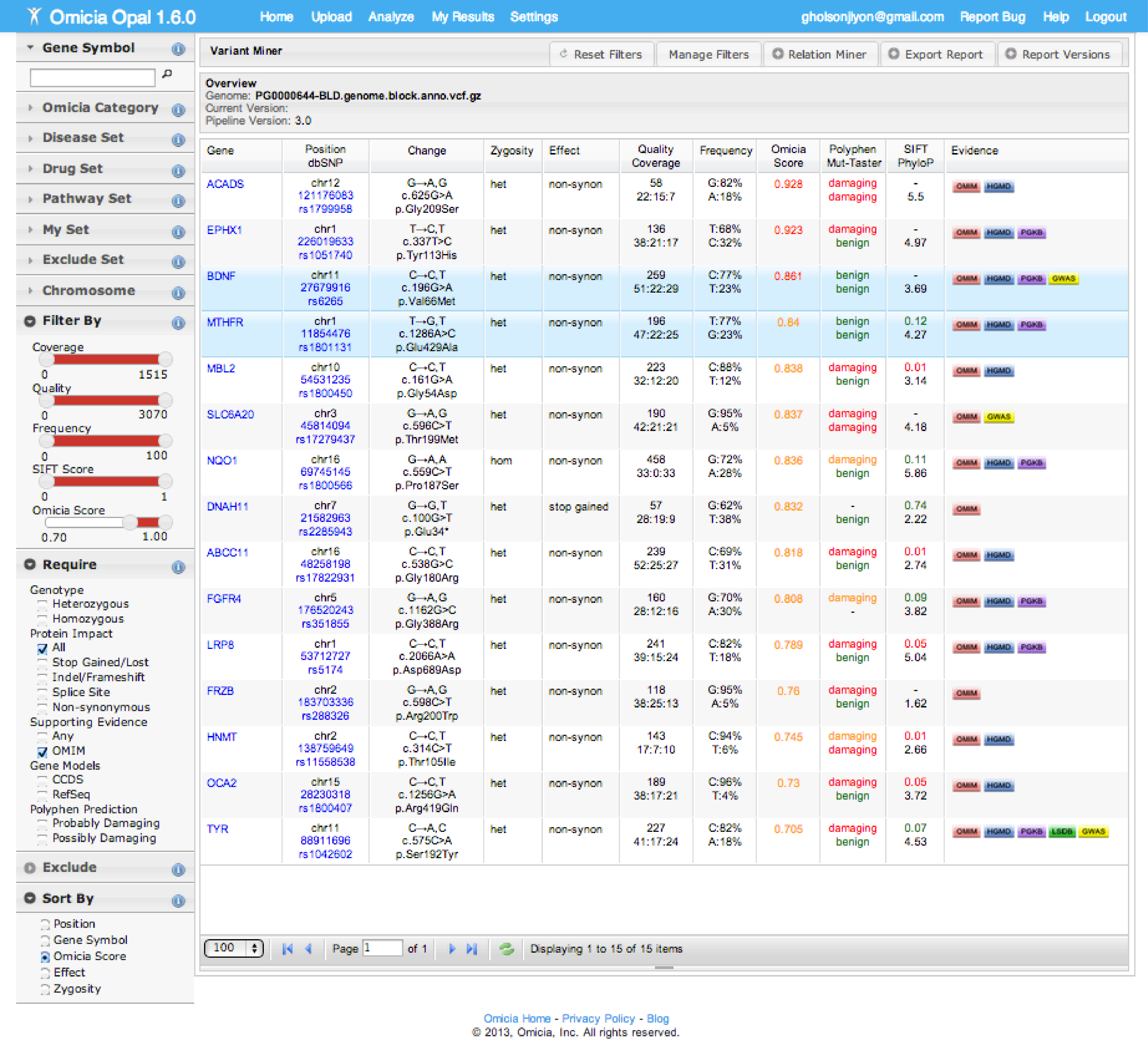

Supplement: Figure S3 [file peerj-01-177-s005.png]

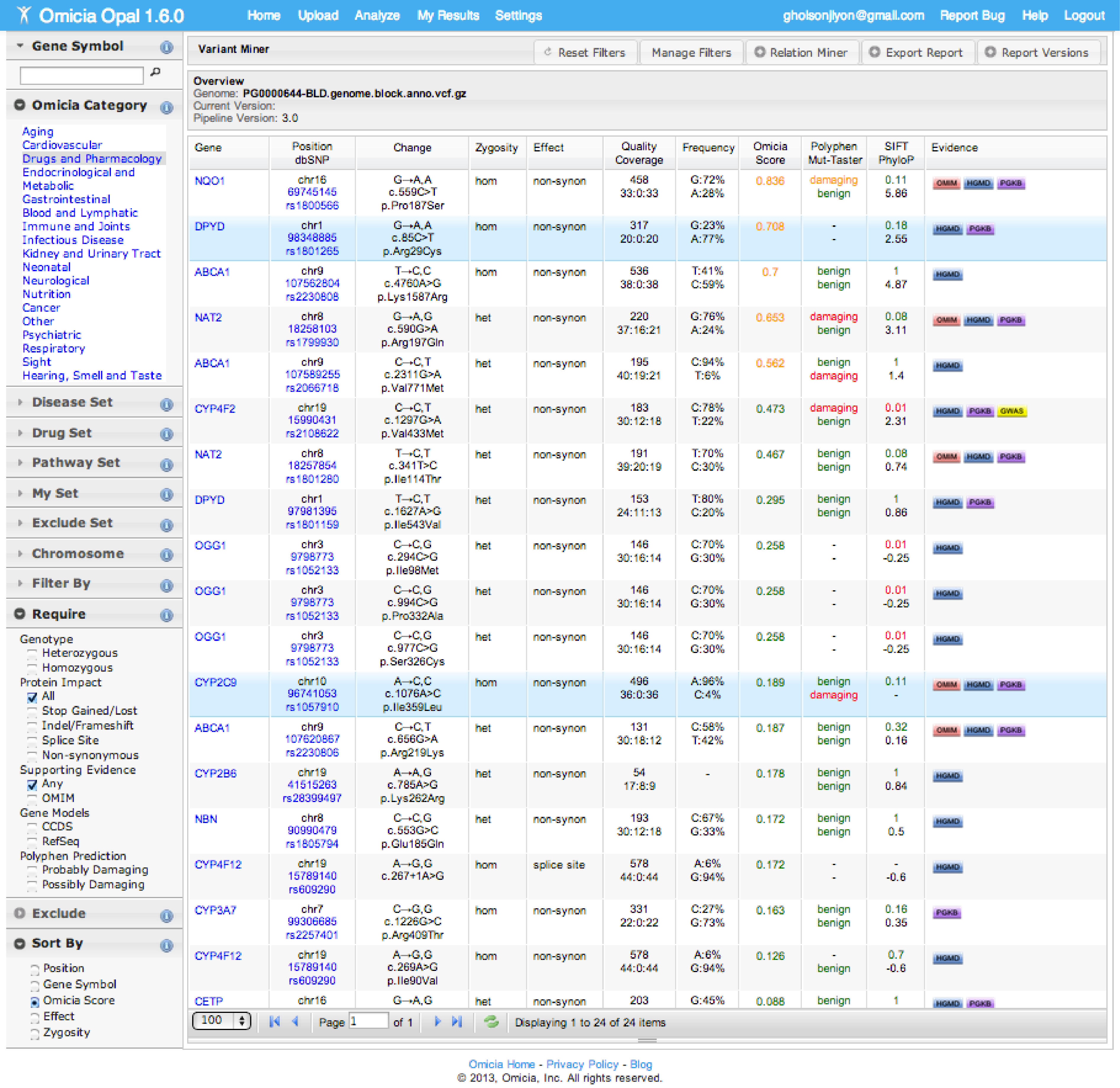

Supplement: Figure S4 [file peerj-01-177-s007.png]

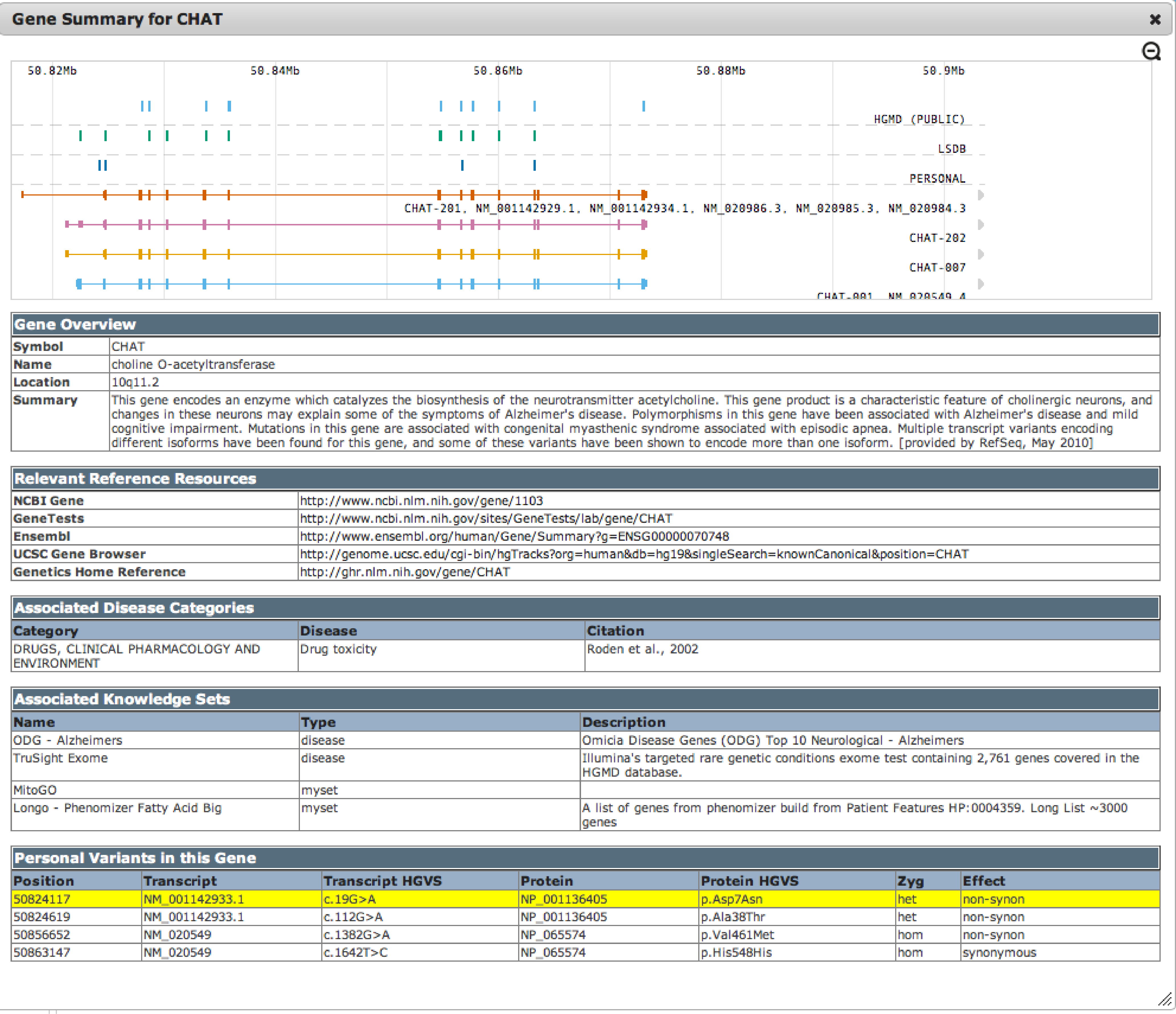

Supplement: Figure S5 — Omicia Opal was used prioritize and identify genetic variations contained within the whole genome sequence of MA that might be of potential clinical relevance to his neuropsychiatric phenotype. In this figure, we highlight the Opal system as being one method by which clinicians can scan genetic data for clinically relevant information in a robust and comprehensive way. We demonstrate the Opal system with one such variant in ChAT, a heterozygous Asp > Asn variation on chromosome 11. [file peerj-01-177-s009.png]
