# Supplementary material for: Integrating precision medicine in the study and clinical treatment of a severely mentally ill person
Source: PeerJ. 2013 Oct 3;1:e177. doi: 10.7717/peerj.177 (PMC3792182; doi:10.7717/peerj.177)
Supplement: File S2 — Clinical interpretation was performed using interpretation guidelines and recommendations from the American College of Medical Genetics. A cumulative total of 1247 variants were detected and evaluated for clinical significance with one single variant being determined as “likely pathogenic”, a p.Arg245Gln change in PHYH. [file peerj-01-177-s004.pdf]

illumina®

Individual Genome Sequence Results

Ordering Physician: Gholson Lyon , MD
Steinmann Institute
10 West Broadway, Suite #820
Salt Lake City UT 84101

Patient Sex	Male
Patient Record Number	PG0000644-BLD
Date Reported	10/14/2012
Indications for Testing	Carrier Screening

Sample Type	Sample Collection Date	Sample Receipt Date
Blood, Peripheral	7/2/2012	7/3/2012

Test - IGS - Individual Genome Sequencing - Wellness

Genome level sequencing was performed and calls made across greater than 90% of the genome. Clinical interpretation was performed using the American College of Medical Genetics recommendations for interpretation on 140 conditions causally associated with 344 genes. The complete list of all interpreted variants for this patient can be found at the end of this report. A list of the 140 conditions and 344 genes can be found on the attached document.

RESULTS

A total of **1247** variants were detected in the subset of genes for this patient. Each variant was evaluated for clinical significance and placed into one of five possible categories for classification, based on the American College of Medical Genetics and Genomics interpretation guidelines as outlined below and described at the end of this report.

Category		Number of Variants	Condition
Clinically Significant in Patient	Pathogenic	0	
	Likely Pathogenic	0	
Carrier Status for Patient	Pathogenic	0	
	Likely Pathogenic	1	Refsum Disease
Variants of Unknown Significance		284	
Likely Benign Variants		349	
Benign Variants		613	

MOLECULAR DIAGNOSTICS REPORT

Clinically Significant in Patient
Variants that are clinically significant increase the individual's risk for a specific disease/disorder that is typically inherited as a dominant condition. Clinical correlation is recommended. The patient's first-degree relatives each have a 50% chance to carry the same variant as the patient. Testing for these at-risk family members should be considered, although the interpretation may be limited by the current understanding of this variant in the case of probably pathogenic variants.

No pathogenic or likely pathogenic variants were found in the 344 genes evaluated that are expected to be clinically significant for the patient. The coverage for these 344 genes is at least 99%. Therefore, significant variants could exist that are not detected with this test. The coverage for each gene is listed in the attached document.

Carrier Status Evaluation
Variants affecting carrier status indicate that an individual does not have the associated disease/disorder but that they may pass the variant to their offspring. These are typically disorders that are inherited in a recessive manner. For some disorders, carriers can manifest symptoms that are typically milder than for affected individuals and they are then referred to as symptomatic carriers. If two carriers of pathogenic variants in the same gene have a child, each child has a 25% chance to be affected when the disease/disorder is inherited in an autosomal recessive fashion. The patient's first-degree relatives each have a 50% chance to be carriers of this same variant. Testing for these at-risk family members should be considered.

Gene	Call	Amino Acid	Interpretation	Associated Condition	Mode of Inheritance
PHYH	c.734G>A	p.Arg245Gln	Likely Pathogenic	Refsum Disease	Autosomal Recessive

<u>Refsum Disease</u>
Refsum disease is an inherited condition that causes vision loss, anosmia, and a variety of other signs and symptoms. The vision loss is caused by retinitis pigmentosa. The first sign of retinitis pigmentosa is usually a loss of night vision, which often becomes apparent in childhood. Over a period of years, the disease disrupts peripheral vision and may eventually lead to blindness. Vision loss and anosmia are seen in almost everyone with Refsum disease, but other signs and symptoms vary. About one-third of affected individuals are born with bone abnormalities of the hands and feet. Features that appear later in life can include progressive myopathy; ataxia; hearing loss; and ichthyosis. Additionally, some people with Refsum disease develop arrhythmia and cardiomyopathies that can be life-threatening.

Jansen et al. (2000) conducted a case-control study of individuals with Refsum disease. In four patients, co-segregation of two missense mutations in the PHYH gene were found - c.530A>G and c.734G>A (Arg245Gln). Two of these patients were compound heterozygotes and two patients were homozygous for these variants. The c.734G>A variant was not found in controls and was not found as an isolated mutation. In silico prediction programs suggest little impact; however, the variant is rare with a 1000 Genomes frequency of ~0.18%. The frequency with which the variant was seen in cases compared to both controls and the 1000 Genomes population frequency is suggestive of pathogenicity.

Variants of Unknown Clinical Significance/Novel Variants
Of the 1247 variants detected in this subset of genes, 284 variants have little or nothing reported about them in the scientific literature, and therefore, are considered Variants of Unknown Significance. This includes

MOLECULAR DIAGNOSTICS REPORT

variants in genes that could be clinically significant or confer carrier status. At this time, the evidence is too weak or contradictory to assess whether the variant is pathogenic or benign. The interpretations of these variants are likely to change as more individuals are sequenced and the community understanding of the effect of the variant improves. A complete list of these variants, the genes in which they were found, and annotation characteristics can be found in the table at the end of this report.

Benign/Likely Benign Variants
Finally, 613 variants categorized as benign and 349 variants categorized as likely benign polymorphisms were also found in these 344 genes. A complete list of these variants, the genes in which they were found, and annotation characteristics can be found in the table at the end of this report.

Evaluation Criteria
As additional evidence may change our understanding of the roles of these variants in their associated conditions, regular review of the implications of these variants should be considered as appropriate. Additional variants that were not included in this interpretation could potentially result in the conditions discussed.
A full list of the citations used to categorize the variants into these groups can be provided upon request.
Criteria for classification:

- Pathogenic: Reported in at least 3 unrelated cases, with control data. Functional or expression evidence suggests deleterious effect on gene function.
- Likely pathogenic: Reported in < 3 cases, or in a single family cohort, with or without control data. Limited or no functional evidence available, but overall biological expectations suggestive of deleterious effect.
- Unknown significance: Little or nothing has been reported on this variant or its effects.
- Likely benign: The variant has been seen in cases, but also in controls. Variant may be present in a high percentage of the population, and may be present in a non-conserved region.
- Benign: Established in the literature as a variant that is not associated with Mendelian (single-gene inherited) disease, or known to have an allele frequency that is far too high to be compatible with the prevalence of disease, mode of inheritance and penetrance patterns known for that condition.

TEST INFORMATION

BACKGROUND:

Clinical interpretation was performed using the American College of Medical Genetics recommendations for interpretation on 140 conditions causally associated with 344 genes. Genome level sequencing was performed and calls made across greater than 90% of the genome. No other variants beyond those contained within the listed genes and conditions were evaluated for possible clinical significance. Therefore, other variants of possible clinical significance may exist within this genome. It is recommended that additional evaluations be performed as appropriate. All calls within these genes were evaluated for evidence of clinical importance including: allele frequency in population studies (dbSNP, 1000 Genomes, etc.), evidence in the scientific literature for likely causation of the condition, and consideration of the likely biological implications of the variant based on its expected characteristics. This assessment represents our current best understanding of the clinical implications of the variants reported. As information within the field increases, this understanding may change and the implications reported here may change. Occasional reassessment of this information is recommended as is appropriate or medically relevant to optimize the medical care of this individual.

METHODOLOGY:

Sequence was generated from DNA that was extracted from peripheral whole blood. The regions of the genome not reported here include regions where the human reference genome has not been completely

MOLECULAR DIAGNOSTICS REPORT

resolved, or where duplications of genetic regions make it impossible to align the fragments accurately. The official reference build 37.1 was used to align the Personal Genome Sequence reported here. (http://www.ncbi.nlm.nih.gov/) The analytical accuracy of these calls is at least 97%. This test was developed and its performance characteristics determined by Illumina Clinical Services Laboratory. It has not been cleared or approved by the U.S. Food and Drug Administration. Pursuant to the requirements of CLIA ’88, this laboratory test has established and verified the test’s accuracy and precision.

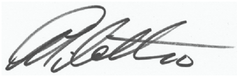

Philip D. Cotter, Ph.D., FACMG
Director, Illumina Clinical Services Laboratory

Signed electronically by Philip Cotter, Ph.D.

REFERENCES:

Jansen GA, Hogenhout EM, Ferdinandusse S, Waterham HR, Ofman R, Jakobs C, Skjeldal OH, Wanders RJ. 2000. Human phytanoyl-CoA hydroxylase: resolution of the gene structure and the molecular basis of Refsum's disease. Hum. Mol. Genet. 9(8):1195-200.

MOLECULAR DIAGNOSTICS REPORT

Interpreted Variants

Gene	dbSNP ID	cDNA	AA	Zygosity	Intepretation
AARS	rs141837805	c.*324G>A		Heterozygous	Unknown Significance
AARS	rs4081753	c.2715T>C	p.Val905Val	Homozygous	Benign
ABCA4		c.5451G	p.Glu1817	Homozygous	Unknown Significance
ABCA4	rs4847281	c.141A>G	p.Pro47Pro	Homozygous	Benign
ABCA4	rs3112831	c.1268A>G	p.His423Arg	Heterozygous	Benign
ABCA4	rs1762114	c.6069T>C	p.Ile2023Ile	Homozygous	Benign
ABCC8		c.579+14C>T		Homozygous	Likely Benign
ABCC8		c.2117-3C>T		Homozygous	Likely Benign
ABCC8	rs1048099	c.207T>C	p.Pro69Pro	Homozygous	Benign
ABCC9		c.574-5C>A		Heterozygous	Benign
ABCC9		c.1164+11A>G		Homozygous	Benign
ABCC9	rs10770865	c.1296T>C	p.Pro432Pro	Homozygous	Benign
ABCC9		c.2199-11T>C		Homozygous	Benign
ACADM		c.*672T>C		Heterozygous	Unknown Significance
ACADM		c.216+10T>C		Heterozygous	Likely Benign
ACADM		c.1161A>G	p.Val387Val	Heterozygous	Likely Benign
ACADM		c.*878T>C		Homozygous	Benign
ACADS		c.625G>A	p.Gly209Ser	Heterozygous	Unknown Significance
ACADS		c.*21G>C		Heterozygous	Unknown Significance
ACADS		c.*527A>G		Heterozygous	Likely Benign
ACADS		c.321T>C	p.Arg107Arg	Heterozygous	Benign
ACADS		c.990C>T	p.Arg330Arg	Heterozygous	Benign
ACADVL		c.1605+6T>C		Heterozygous	Benign
ACTC1		c.*472T>C		Heterozygous	Unknown Significance
ACTC1		c.*388G>A		Heterozygous	Likely Benign
ACTC1		c.*1682A>G		Heterozygous	Likely Benign
ACTC1		c.*2090A>G		Heterozygous	Likely Benign
ACTC1		c.*1039G>A		Homozygous	Benign
ACTN2		c.*447C>G		Homozygous	Likely Benign
ACTN2		c.*748A>G		Homozygous	Likely Benign
ACTN2	rs1341864	c.351T>C	p.Ile117Ile	Homozygous	Benign
ACTN2	rs1341863	c.378C>T	p.Asn126Asn	Homozygous	Benign
ACTN2		c.877-8C>G		Homozygous	Benign
ACTN2		c.*526T>C		Homozygous	Benign
ACVRL1		c.*1662A>T		Heterozygous	Unknown Significance
ACVRL1		c.313+11C>T		Homozygous	Likely Benign
ACVRL1		c.*1246T>C		Homozygous	Benign
ADA	rs394105	c.36G>A	p.Val12Val	Homozygous	Benign
ADAMTS2		c.858C>T	p.His286His	Heterozygous	Likely Benign

Illumina Clinical Services Laboratory PG0000644-BLD

MOLECULAR DIAGNOSTICS REPORT

Gene	dbSNP ID	cDNA	AA	Zygosity	Intepretation
ADAMTS2	rs2278221	c.1194C>T	p.Asp398Asp	Heterozygous	Likely Benign
ADAMTS2	rs1054480	c.3529C>T	p.Pro1177Ser	Heterozygous	Likely Benign
ADAMTS2		c.*1586A>G		Heterozygous	Likely Benign
ADAMTS2		c.*2396C>T		Heterozygous	Likely Benign
ADAMTS2		c.*2951G>A		Heterozygous	Likely Benign
ADAMTS2		c.*2952T>A		Heterozygous	Likely Benign
ADAMTS2	rs423552	c.786G>A	p.Ala262Ala	Homozygous	Benign
ADAMTS2	rs35462609	c.936C>T	p.Asn312Asn	Heterozygous	Benign
ADAMTS2		c.*212A>C		Heterozygous	Benign
ADAMTS2		c.*842T>C		Heterozygous	Benign
AGA		c.*183A>C		Homozygous	Likely Benign
AGA	rs2228119	c.446C>G	p.Thr149Ser	Homozygous	Benign
AKAP9		c.1389G>T	p.Met463Ile	Heterozygous	Unknown Significance
AKAP9		c.6888A>G	p.Gln2296Gln	Heterozygous	Unknown Significance
AKAP9		c.8375A>G	p.Asn2792Ser	Heterozygous	Unknown Significance
AKAP9		c.3504A>G	p.Glu1168Glu	Heterozygous	Likely Benign
AKAP9		c.5778C>T	p.Gly1926Gly	Heterozygous	Likely Benign
AKAP9		c.6945+8C>T		Heterozygous	Likely Benign
AKAP9		c.8665C>T	p.Leu2889Leu	Heterozygous	Likely Benign
AKAP9		c.9145C>T	p.Leu3049Leu	Heterozygous	Likely Benign
AKAP9		c.10426A>C	p.Arg3476Arg	Heterozygous	Likely Benign
AKAP9		c.-124G>C		Heterozygous	Benign
AKAP9	rs1989779	c.3075C>T	p.Thr1025Thr	Homozygous	Benign
AKAP9	rs1063242	c.8935C>T	p.Pro2979Ser	Homozygous	Benign
ALS2		c.4119A>G	p.Ile1373Met	Heterozygous	Unknown Significance
ALS2		c.20+7T>C		Heterozygous	Likely Benign
ALS2	rs3219156	c.1102G>A	p.Val368Met	Homozygous	Benign
ALS2	rs2276615	c.2466G>A	p.Val822Val	Homozygous	Benign
ALS2	rs3219168	c.4015C>T	p.Leu1339Leu	Homozygous	Benign
ANK1		c.3224C	p.Thr1075	Homozygous	Unknown Significance
ANK1		c.4385C>T	p.Ala1462Val	Heterozygous	Unknown Significance
ANK1		c.4506C>T	p.Arg1502Arg	Heterozygous	Unknown Significance
ANK1	rs2304871	c.315C>T	p.Asn105Asn	Heterozygous	Likely Benign
ANK1		c.4101C>T	p.Ala1367Ala	Heterozygous	Likely Benign
ANK1		c.5479-3T>C		Homozygous	Benign
ANK2		c.3579C>T	p.Arg1193Arg	Heterozygous	Likely Benign
ANK2		c.3893+14G>T		Heterozygous	Likely Benign
ANK2	rs3733615	c.7110A>G	p.Gln2370Gln	Heterozygous	Likely Benign
ANK2		c.8503C>T	p.Pro2835Ser	Heterozygous	Likely Benign
ANK2	rs2293324	c.11673T>C	p.His3891His	Heterozygous	Likely Benign
ANO5	rs35827261	c.*3121A>G		Heterozygous	Unknown Significance

Illumina Clinical Services Laboratory PG0000644-BLD

MOLECULAR DIAGNOSTICS REPORT

Gene	dbSNP ID	cDNA	AA	Zygosity	Intepretation
ANO5	rs7481951	c.966A>T	p.Leu322Phe	Heterozygous	Likely Benign
ANO5		c.*496A>G		Heterozygous	Likely Benign
ANO5		c.-136G>C		Homozygous	Benign
ANO5	rs4312063	c.267T>C	p.Asp89Asp	Homozygous	Benign
ANO5		c.*1286G>T		Heterozygous	Benign
ANO5		c.*3178C>G		Heterozygous	Benign
APC		c.*1098T>C		Homozygous	Likely Benign
APC		c.*1556C>G		Homozygous	Likely Benign
APC		c.1458T>C	p.Tyr486Tyr	Homozygous	Benign
APC	rs351771	c.1635G>A	p.Ala545Ala	Homozygous	Benign
APC	rs41115	c.4479G>A	p.Thr1493Thr	Homozygous	Benign
APC	rs42427	c.5034G>A	p.Gly1678Gly	Homozygous	Benign
APC	rs866006	c.5268T>G	p.Ser1756Ser	Homozygous	Benign
APC	rs459552	c.5465T>A	p.Val1822Asp	Homozygous	Benign
APC	rs465899	c.5880G>A	p.Pro1960Pro	Homozygous	Benign
APC		c.*1753G>A		Homozygous	Benign
APOB		c.10294C	p.Gln3432	Homozygous	Unknown Significance
APOB	rs1041968	c.6936C>T		Heterozygous	Likely Benign
APOB	rs693	c.7545C>T		Heterozygous	Likely Benign
APOB	rs679899	c.1853C>T		Heterozygous	Benign
APOB	rs568413	c.4265A>G		Homozygous	Benign
APOB	rs584542	c.6937A>G		Homozygous	Benign
APOB	rs676210	c.8216C>T		Heterozygous	Benign
APOB	rs1042034	c.13013G>A		Heterozygous	Benign
APP		c.-111G>C		Heterozygous	Unknown Significance
ARSA	rs743616	c.1178C>G	p.Thr393Ser	Homozygous	Likely Benign
ARSA		c.*682G>C		Homozygous	Likely Benign
ARSA		c.*855G>A		Homozygous	Likely Benign
ARSA		c.*919A>G		Homozygous	Benign
ARSA		c.*1351C>G		Homozygous	Benign
ARSB		c.-958A>G		Homozygous	Unknown Significance
ARSB		c.-906C>G		Homozygous	Unknown Significance
ARSB		c.-564A>G		Homozygous	Unknown Significance
ARSB		c.1191G>A	p.Pro397Pro	Heterozygous	Likely Benign
ARSB		c.*2022T>G		Homozygous	Likely Benign
ARSB		c.-302A>G		Homozygous	Benign
ARSB		c.*2975G>T		Homozygous	Benign
ASAH1		c.*926A>C		Homozygous	Likely Benign
ASAH1	rs10103355	c.737T>C	p.Val246Ala	Homozygous	Benign
ASAH1		c.*1073C>T		Homozygous	Benign
ASPA	rs12948217	c.693C>T	p.Tyr231Tyr	Homozygous	Likely Benign
ASPA		c.*139C>A		Homozygous	Benign
ASS1		c.-323G>T		Heterozygous	Unknown Significance

Illumina Clinical Services Laboratory PG0000644-BLD

MOLECULAR DIAGNOSTICS REPORT

Gene	dbSNP ID	cDNA	AA	Zygosity	Intepretation
ATP7B		c.287G	p.Gly96	Homozygous	Unknown Significance
ATP7B		c.2623G	p.Gly875	Homozygous	Unknown Significance
ATP7B	rs1801248	c.3045G>A	p.Leu1015Leu	Heterozygous	Unknown Significance
ATP7B		c.3903+6C>T		Homozygous	Likely Benign
ATP7B		c.*1172G>A		Homozygous	Likely Benign
ATP7B		c.-75C>A		Homozygous	Benign
ATP7B	rs1801243	c.1216T>G	p.Ser406Ala	Homozygous	Benign
ATP7B	rs1801244	c.1366G>C	p.Val456Leu	Homozygous	Benign
ATP7B	rs1061472	c.2495A>G	p.Lys832Arg	Homozygous	Benign
ATP7B	rs732774	c.2855G>A	p.Arg952Lys	Homozygous	Benign
ATP7B	rs1801249	c.3419T>C	p.Val1140Ala	Homozygous	Benign
BCKDHB		c.*731C>T		Heterozygous	Likely Benign
BCKDHB		c.*121G>A		Homozygous	Benign
BCKDHB		c.*293T>C		Homozygous	Benign
BCKDHB		c.*304G>A		Homozygous	Benign
BCKDHB		c.*345C>T		Homozygous	Benign
BCKDHB		c.*395G>T		Homozygous	Benign
BCKDHB		c.*627T>C		Homozygous	Benign
BCKDHB		c.*789C>T		Homozygous	Benign
BCKDHB		c.*805T>C		Homozygous	Benign
BCKDHB		c.*994G>A		Homozygous	Benign
BCKDHB		c.*1142C>G		Homozygous	Benign
BCKDHB		c.*1241A>G		Homozygous	Benign
BCKDHB		c.*1444T>C		Homozygous	Benign
BCKDHB		c.*1778A>G		Homozygous	Benign
BCKDHB		c.*1810T>C		Homozygous	Benign
BCKDHB		c.*1875G>A		Homozygous	Benign
BCKDHB		c.*2271G>A		Homozygous	Benign
BCKDHB		c.*2282C>T		Homozygous	Benign
BEST1		c.201G>C	p.Leu67Leu	Heterozygous	Unknown Significance
BEST1		c.-221T>C		Heterozygous	Benign
BEST1	rs1800007	c.109T>C	p.Leu37Leu	Heterozygous	Benign
BRCA1	rs4986850	c.2077G>A	p.Asp693Asn	Heterozygous	Unknown Significance
BRCA1	rs1799949	c.2082C>T	p.Ser694Ser	Heterozygous	Likely Benign
BRCA1	rs16940	c.2311T>C	p.Leu771Leu	Heterozygous	Likely Benign
BRCA1	rs16941	c.3113A>G	p.Glu1038Gly	Heterozygous	Likely Benign
BRCA1	rs16942	c.3548A>G	p.Lys1183Arg	Heterozygous	Likely Benign
BRCA1	rs1060915	c.4308T>C	p.Ser1436Ser	Heterozygous	Likely Benign
BRCA1	rs1799966	c.4837A>G	p.Ser1613Gly	Heterozygous	Likely Benign
BRCA1		c.*421G>T		Heterozygous	Likely Benign
BRCA1	rs799917	c.2612C>T	p.Pro871Leu	Heterozygous	Benign
BRCA2		c.865A>C		Heterozygous	Unknown Significance

Illumina Clinical Services Laboratory PG0000644-BLD

MOLECULAR DIAGNOSTICS REPORT

Gene	dbSNP ID	cDNA	AA	Zygosity	Intepretation
BRCA2		c.1365A>G		Heterozygous	Unknown Significance
BRCA2		c.2229T>C		Heterozygous	Unknown Significance
BRCA2		c.2971A>G		Heterozygous	Unknown Significance
BRCA2		c.*105A>C		Heterozygous	Unknown Significance
BRCA2	rs543304	c.3807T>C		Heterozygous	Benign
BRCA2	rs206075	c.4563A>G		Homozygous	Benign
BRCA2	rs206076	c.6513G>C		Homozygous	Benign
BRCA2	rs169547	c.7397T>C		Homozygous	Benign
BRCA2		c.7806-14T>C		Heterozygous	Benign
BRIP1		c.*2090G>C		Heterozygous	Unknown Significance
BRIP1		c.*3488A>T		Heterozygous	Likely Benign
BRIP1	rs4986765	c.2637A>G	p.Glu879Glu	Heterozygous	Benign
BRIP1	rs4986764	c.2755T>C	p.Ser919Pro	Heterozygous	Benign
BRIP1	rs4986763	c.3411T>C	p.Tyr1137Tyr	Heterozygous	Benign
BRIP1		c.*483C>T		Heterozygous	Benign
BRIP1		c.*3514T>G		Heterozygous	Benign
BSND	rs33938617	c.924G>A	p.Pro308Pro	Heterozygous	Likely Benign
BSND		c.*24A>C		Heterozygous	Likely Benign
BSND		c.-117T>C		Homozygous	Benign
BSND		c.-70C>G		Homozygous	Benign
CACNA1C	rs216008	c.3786C>T	p.Phe1262Phe	Heterozygous	Likely Benign
CACNA1C	rs1544514	c.522G>A	p.Ala174Ala	Heterozygous	Benign
CACNA1C	rs56180838	c.4038C>T	p.Ile1346Ile	Heterozygous	Benign
CACNA1C	rs1051375	c.5361G>A	p.Thr1787Thr	Homozygous	Benign
CACNA1C		c.*284C>T		Heterozygous	Benign
CACNA1C		c.*4273A>G		Homozygous	Benign
CACNA1C		c.*4831T>A		Homozygous	Benign
CACNA1C		c.*5970A>C		Homozygous	Benign
CACNB2		c.-253G>A		Heterozygous	Unknown Significance
CACNB2		c.*10G>T		Homozygous	Unknown Significance
CACNB2		c.*608T>A		Heterozygous	Unknown Significance
CACNB2		c.*609A>T		Heterozygous	Unknown Significance
CACNB2		c.892+7C>T		Heterozygous	Likely Benign
CACNB2	rs2228645	c.1539C>T	p.Tyr513Tyr	Heterozygous	Likely Benign
CACNB2		c.*1965C>T		Heterozygous	Likely Benign
CACNB2		c.*2016T>C		Homozygous	Benign
CAPN3		c.96T>C	p.Thr32Thr	Heterozygous	Unknown Significance
CAPN3		c.*134C>T		Homozygous	Benign
CDH1		c.1937-13T>C		Heterozygous	Unknown Significance

Illumina Clinical Services Laboratory PG0000644-BLD

MOLECULAR DIAGNOSTICS REPORT

Gene	dbSNP ID	cDNA	AA	Zygosity	Intepretation
CDH1		c.*1120T>C		Heterozygous	Unknown Significance
CDH1		c.48+6C>T		Homozygous	Benign
CDH1		c.2076T>C	p.Ala692Ala	Homozygous	Benign
CDH23		c.1469G>C	p.Gly490Ala	Heterozygous	Unknown Significance
CDH23		c.5411G>A	p.Arg1804Gln	Heterozygous	Unknown Significance
CDH23		c.5503-10A>G		Heterozygous	Unknown Significance
CDH23		c.6130G>A	p.Glu2044Lys	Heterozygous	Unknown Significance
CDH23		c.7073G>A	p.Arg2358Gln	Heterozygous	Unknown Significance
CDH23		c.7139C>T	p.Pro2380Leu	Heterozygous	Unknown Significance
CDH23		c.8895C>T	p.Pro2965Pro	Heterozygous	Unknown Significance
CDH23		c.9077+8G>A		Heterozygous	Unknown Significance
CDH23		c.9319+11G>A		Heterozygous	Unknown Significance
CDH23		c.5100C>T	p.Tyr1700Tyr	Heterozygous	Likely Benign
CDH23		c.7572G>A	p.Ala2524Ala	Heterozygous	Likely Benign
CDH23	rs3752752	c.2316T>C	p.Asn772Asn	Heterozygous	Benign
CDH23	rs3752751	c.2388T>C	p.Asp796Asp	Heterozygous	Benign
CDH23	rs1227065	c.4051A>G	p.Asn1351Asp	Heterozygous	Benign
CDH23		c.4723G>A	p.Ala1575Thr	Heterozygous	Benign
CDH23		c.*349A>G		Heterozygous	Benign
CDK4		c.*521G>A		Heterozygous	Unknown Significance
CERKL		c.*1159C>T		Homozygous	Likely Benign
CERKL	rs1473295	c.156C>T	p.Phe52Phe	Heterozygous	Benign
CERKL		c.1133+13T>C		Homozygous	Benign
CERKL	rs10180793	c.1506C>T	p.Asp502Asp	Homozygous	Benign
CERKL		c.*121C>T		Homozygous	Benign
CERKL		c.*880G>A		Homozygous	Benign
CERKL		c.*1345G>A		Homozygous	Benign
CFTR		c.869+11C>T		Heterozygous	Unknown Significance
CFTR		c.1365G>T	p.Ala455Ala	Heterozygous	Unknown Significance
CFTR	rs1800131	c.3897A>G	p.Thr1299Thr	Heterozygous	Unknown Significance
CFTR	rs213950	c.1408G>A	p.Val470Met	Heterozygous	Benign
CHMP2B		c.*1589G>A		Heterozygous	Likely Benign
CHMP2B	rs11540913	c.312T>C	p.Thr104Thr	Homozygous	Benign
CLDN14	rs219780	c.687G>A	p.Thr229Thr	Heterozygous	Unknown Significance
CLDN14		c.243C>T	p.Arg81Arg	Heterozygous	Likely Benign
CLN6		c.*817C>T		Heterozygous	Likely Benign

Illumina Clinical Services Laboratory PG0000644-BLD

MOLECULAR DIAGNOSTICS REPORT

Gene	dbSNP ID	cDNA	AA	Zygosity	Intepretation
CNGA1	rs224801	c.-117C>T		Homozygous	Likely Benign
CNGB1		c.*1228C>G		Homozygous	Unknown Significance
CNGB1	rs17821448	c.327C>T	p.Gly109Gly	Heterozygous	Likely Benign
CNGB1		c.1122-15C>T		Heterozygous	Likely Benign
CNGB1		c.2193C>T	p.Asn731Asn	Heterozygous	Likely Benign
CNGB1		c.2218-12C>T		Heterozygous	Likely Benign
CNGB1		c.3462+7T>C		Homozygous	Likely Benign
CNGB1		c.*919G>A		Homozygous	Likely Benign
CNGB1	rs13336595	c.299G>A	p.Arg100His	Heterozygous	Benign
CNGB1		c.2635-10C>T		Heterozygous	Benign
CNGB1	rs413562	c.2664C>G	p.Ala888Ala	Heterozygous	Benign
COCH		c.*27A>T		Heterozygous	Unknown Significance
COCH	rs1045644	c.1055C>G	p.Thr352Ser	Homozygous	Likely Benign
COL11A2		c.826G>A	p.Glu276Lys	Heterozygous	Likely Benign
COL11A2		c.877-4T>A		Heterozygous	Likely Benign
COL11A2		c.3384C>T	p.Pro1128Pro	Heterozygous	Likely Benign
COL11A2		c.1360-7A>C		Homozygous	Benign
COL11A2		c.2628+3G>A		Heterozygous	Benign
COL11A2	rs2229785	c.2700T>C	p.Asp900Asp	Heterozygous	Benign
COL11A2		c.3150+15A>C		Heterozygous	Benign
COL11A2	rs1799910	c.3174G>A	p.Pro1058Pro	Heterozygous	Benign
COL11A2		c.3313-11C>T		Heterozygous	Benign
COL1A2	rs412777	c.1446A>C	p.Pro482Pro	Heterozygous	Likely Benign
COL1A2		c.936+14C>T		Heterozygous	Benign
COL1A2		c.937-3C>T		Heterozygous	Benign
COL1A2	rs42524	c.1645C>G	p.Pro549Ala	Heterozygous	Benign
COL3A1		c.2244T>C		Heterozygous	Likely Benign
COL3A1	rs1516446	c.4059T>G		Homozygous	Benign
COL5A1	rs3827848	c.4122G>A	p.Thr1374Thr	Heterozygous	Unknown Significance
COL5A1	rs77176843	c.4230+6G>A		Heterozygous	Unknown Significance
COL5A1		c.738C>T	p.Thr246Thr	Heterozygous	Likely Benign
COL5A1		c.4176+9T>G		Heterozygous	Likely Benign
COL5A1	rs3811146	c.4482G>C	p.Pro1494Pro	Heterozygous	Likely Benign
COL5A1		c.*267C>T		Homozygous	Likely Benign
COL5A1		c.*733C>A		Homozygous	Likely Benign
COL5A1		c.-247T>G		Homozygous	Benign
COL5A1		c.1432-5T>C		Heterozygous	Benign
COL5A1		c.2952+11A>T		Heterozygous	Benign
COL5A1		c.*83C>T		Homozygous	Benign
COL5A1		c.*1125A>T		Homozygous	Benign
COL5A1		c.*2395G>T		Homozygous	Benign
COL5A1		c.*2501T>C		Homozygous	Benign
COL5A2	rs4128539	c.315C>A	p.Thr105Thr	Homozygous	Benign
COL5A2	rs2229495	c.1311A>G	p.Pro437Pro	Homozygous	Benign

Illumina Clinical Services Laboratory PG0000644-BLD

MOLECULAR DIAGNOSTICS REPORT

Gene	dbSNP ID	cDNA	AA	Zygosity	Intepretation
COL5A2	rs6434312	c.3411T>C	p.Gly1137Gly	Homozygous	Benign
COL6A1		c.2549G>A	p.Arg850His	Heterozygous	Unknown Significance
COL6A1		c.1957-11C>T		Heterozygous	Likely Benign
COL6A1		c.2667G>A	p.Ala889Ala	Heterozygous	Likely Benign
COL6A1		c.2796C>T	p.Ser932Ser	Heterozygous	Likely Benign
COL6A1		c.428+14A>G		Homozygous	Benign
COL6A1	rs1980982	c.1095T>C	p.Gly365Gly	Heterozygous	Benign
COL6A1		c.1956+15C>T		Heterozygous	Benign
COL6A1		c.2434+15A>G		Homozygous	Benign
COL6A1		c.*260A>G		Homozygous	Benign
COL6A1		c.*419A>G		Homozygous	Benign
COL6A3	rs1131296	c.9206C>T	p.Thr3069Ile	Heterozygous	Unknown Significance
COL6A3	rs4433949	c.7929G>A	p.Ala2643Ala	Heterozygous	Likely Benign
COL6A3	rs3790993	c.6855G>C	p.Gly2285Gly	Heterozygous	Benign
COL6A3	rs6728818	c.8780T>C	p.Met2927Thr	Heterozygous	Benign
COL6A3	rs2270669	c.9034G>C	p.Ala3012Pro	Heterozygous	Benign
CPS1		c.*438T>G		Heterozygous	Likely Benign
CPS1		c.*692C>G		Heterozygous	Likely Benign
CPS1	rs1047883	c.1030A>G	p.Thr344Ala	Homozygous	Benign
CPS1	rs2229589	c.1032C>T	p.Thr344Thr	Homozygous	Benign
CPS1	rs2287599	c.2679C>G	p.Gly893Gly	Homozygous	Benign
CPT2		c.1102G>A	p.Val368Ile	Homozygous	Benign
CRB1	rs3902057	c.1410A>G	p.Leu470Leu	Homozygous	Unknown Significance
CRX		c.100+12C>T		Heterozygous	Unknown Significance
CRX		c.*1046C>T		Heterozygous	Unknown Significance
CRX		c.*1289G>A		Heterozygous	Unknown Significance
CRX		c.*2380C>T		Heterozygous	Unknown Significance
CRX		c.*400A>T		Heterozygous	Likely Benign
CRX		c.*401A>C		Heterozygous	Likely Benign
CRX		c.*579T>C		Heterozygous	Likely Benign
CRX		c.*591G>C		Heterozygous	Likely Benign
CRX		c.*682C>T		Heterozygous	Likely Benign
CRX		c.*966G>C		Heterozygous	Likely Benign
CRX		c.*1346G>A		Heterozygous	Likely Benign
CRX		c.*2106C>T		Heterozygous	Likely Benign
CRX		c.*2559G>C		Heterozygous	Likely Benign
CRX		c.*2704C>T		Heterozygous	Likely Benign
CRX		c.*2717G>T		Heterozygous	Likely Benign
CRX		c.*2937T>C		Heterozygous	Likely Benign
CRX		c.*3017C>T		Heterozygous	Likely Benign
CRX		c.*3279C>T		Heterozygous	Likely Benign
CRX		c.*3301T>C		Heterozygous	Likely Benign

MOLECULAR DIAGNOSTICS REPORT

Gene	dbSNP ID	cDNA	AA	Zygosity	Intepretation
CRX		c.*1455T>A		Heterozygous	Benign
CRX		c.*2183T>C		Heterozygous	Benign
CTF1		c.*704T>C		Homozygous	Benign
CTNS		c.*737T>A		Heterozygous	Likely Benign
CTNS	rs161400	c.779C>T	p.Thr260Ile	Homozygous	Benign
CTNS		c.*738T>G		Homozygous	Benign
CTNS		c.*1440A>G		Homozygous	Benign
CTNS		c.*2306T>C		Homozygous	Benign
CTSA		c.-172A>C		Heterozygous	Unknown Significance
CTSA		c.-322G>A		Heterozygous	Likely Benign
CTSA		c.1002+7G>A		Heterozygous	Benign
CTSA		c.1142+10C>T		Heterozygous	Benign
DBT	rs115442245	c.*5919A>G		Heterozygous	Unknown Significance
DBT		c.*9082C>T		Heterozygous	Unknown Significance
DBT	rs12021720	c.1150A>G	p.Ser384Gly	Homozygous	Likely Benign
DBT		c.*5317T>C		Heterozygous	Likely Benign
DBT		c.*3233C>T		Homozygous	Benign
DBT		c.*5150A>G		Homozygous	Benign
DBT		c.*5546G>A		Homozygous	Benign
DBT		c.*6228A>T		Homozygous	Benign
DES	rs1058261	c.828C>T	p.Asp276Asp	Heterozygous	Likely Benign
DES	rs12920	c.1014G>C	p.Leu338Leu	Heterozygous	Likely Benign
DES	rs1058284	c.1104G>A	p.Ala368Ala	Heterozygous	Likely Benign
DES	rs1318299	c.75A>G	p.Pro25Pro	Homozygous	Benign
DES	rs2017800	c.93T>C	p.Ser31Ser	Homozygous	Benign
DFNB31		c.1318G>A		Heterozygous	Unknown Significance
DFNB31		c.1627-12G>A		Heterozygous	Unknown Significance
DFNB31		c.2388C>A		Heterozygous	Unknown Significance
DFNB31		c.-510G>A		Heterozygous	Likely Benign
DFNB31		c.-391C>A		Heterozygous	Likely Benign
DFNB31	rs10817610	c.1091G>A		Homozygous	Benign
DFNB31	rs4979387	c.1353T>C		Heterozygous	Benign
DFNB31	rs942519	c.1838T>C		Heterozygous	Benign
DFNB31	rs6478078	c.2256C>G		Homozygous	Benign
DFNB31	rs2274159	c.2348T>C		Heterozygous	Benign
DFNB59		c.793C>G	p.Arg265Gly	Heterozygous	Unknown Significance
DHCR7		c.-23T>C		Heterozygous	Unknown Significance
DHCR7	rs4316537	c.231C>T	p.Thr77Thr	Heterozygous	Unknown Significance
DHCR7	rs949177	c.438T>C	p.Asn146Asn	Homozygous	Likely Benign
DHCR7		c.626+15G>A		Homozygous	Likely Benign
DHCR7		c.*480C>T		Homozygous	Likely Benign

Illumina Clinical Services Laboratory PG0000644-BLD

MOLECULAR DIAGNOSTICS REPORT

Gene	dbSNP ID	cDNA	AA	Zygosity	Intepretation
DHCR7	rs1790334	c.207T>C	p.Thr69Thr	Homozygous	Benign
DHCR7	rs760241	c.1158T>C	p.Asp386Asp	Homozygous	Benign
DHCR7		c.*643C>T		Homozygous	Benign
DHCR7		c.*734A>G		Homozygous	Benign
DMD	rs228406	c.2645A>G		Homozygous	Benign
DMD	rs1801187	c.5234G>A		Homozygous	Benign
DMD	rs1800280	c.8810G>A		Homozygous	Benign
DMD		c.9649+15T>C		Homozygous	Benign
DSP		c.273+10C>T		Heterozygous	Unknown Significance
DSP	rs2806234	c.741T>G	p.Ala247Ala	Homozygous	Benign
DSP	rs2076304	c.2091A>G	p.Gly697Gly	Homozygous	Benign
DSP	rs1016835	c.2631G>A	p.Arg877Arg	Homozygous	Benign
DSP	rs2744380	c.8472G>C	p.Gly2824Gly	Homozygous	Benign
DTNA	rs117571555	c.210G>A	p.Leu70Leu	Heterozygous	Unknown Significance
DTNA		c.*172C>T		Heterozygous	Unknown Significance
DTNA		c.*174A>G		Heterozygous	Unknown Significance
DTNA		c.*310C>T		Heterozygous	Unknown Significance
DTNA		c.*2126C>T		Heterozygous	Unknown Significance
DTNA		c.1653+14G>A		Heterozygous	Likely Benign
DYSF		c.1351A>G	p.Met451Val	Heterozygous	Unknown Significance
DYSF		c.6204+15C>T		Heterozygous	Unknown Significance
DYSF		c.1353+13C>T		Homozygous	Likely Benign
DYSF	rs17718530	c.5859A>C	p.Pro1953Pro	Heterozygous	Likely Benign
DYSF	rs2303596	c.1827T>C	p.Asp609Asp	Homozygous	Benign
DYSF	rs2288355	c.2583A>T	p.Ser861Ser	Heterozygous	Benign
DYSF	rs2303606	c.4008C>A	p.Ile1336Ile	Heterozygous	Benign
EGR2		c.*288G>A		Heterozygous	Unknown Significance
EGR2	rs224083	c.627G>A	p.Pro209Pro	Homozygous	Benign
ENG		c.1029C>T	p.Thr343Thr	Heterozygous	Unknown Significance
ENG	rs142803546	c.1095C>T	p.Asp365Asp	Heterozygous	Unknown Significance
ENG		c.-324A>G		Homozygous	Benign
ESRRB		c.*1068C>T		Heterozygous	Unknown Significance
ESRRB		c.*643T>C		Heterozygous	Likely Benign
ESRRB	rs2361293	c.885T>C	p.Tyr295Tyr	Homozygous	Benign
EYA4		c.829G>A	p.Gly277Ser	Heterozygous	Unknown Significance
EYA4		c.*2302G>A		Heterozygous	Unknown Significance

Illumina Clinical Services Laboratory

PG0000644-BLD

MOLECULAR DIAGNOSTICS REPORT

Gene	dbSNP ID	cDNA	AA	Zygosity	Intepretation
EYA4		c.*3014T>C		Heterozygous	Unknown Significance
EYA4		c.*23C>T		Homozygous	Likely Benign
EYA4		c.*1264T>A		Homozygous	Benign
EYA4		c.*1434A>G		Homozygous	Benign
EYS		c.1922A>T	p.Glu641Val	Heterozygous	Unknown Significance
EYS		c.3787A>G	p.Ile1263Val	Heterozygous	Unknown Significance
EYS		c.3973C>G	p.Gln1325Glu	Heterozygous	Unknown Significance
EYS		c.4081A>G	p.Ile1361Val	Heterozygous	Unknown Significance
EYS		c.4352T>C	p.Ile1451Thr	Heterozygous	Unknown Significance
EYS		c.4543C>T	p.Arg1515Trp	Heterozygous	Unknown Significance
EYS		c.4549A>G	p.Ser1517Gly	Heterozygous	Unknown Significance
EYS		c.5617C>G	p.Leu1873Val	Heterozygous	Unknown Significance
EYS	rs12193967	c.359C>T	p.Thr120Met	Homozygous	Likely Benign
EYS		c.3444-5C>T		Heterozygous	Likely Benign
EYS		c.3906C>T	p.His1302His	Heterozygous	Likely Benign
EYS		c.3936A>G	p.Thr1312Thr	Heterozygous	Likely Benign
EYS		c.4026C>T	p.Ser1342Ser	Heterozygous	Likely Benign
EYS		c.4593G>A	p.Glu1531Glu	Heterozygous	Likely Benign
EYS	rs974110	c.1146T>C	p.Asn382Asn	Homozygous	Benign
EYS	rs9345601	c.1809C>T	p.Val603Val	Heterozygous	Benign
EYS	rs9342464	c.1891G>A	p.Gly631Ser	Heterozygous	Benign
EYS	rs9294631	c.2555T>C	p.Leu852Pro	Homozygous	Benign
EYS	rs624851	c.4256T>C	p.Leu1419Ser	Homozygous	Benign
F2		c.494C>T	p.Thr165Met	Heterozygous	Unknown Significance
F5		c.1242A		Homozygous	Unknown Significance
F5	rs4524	c.2573A>G		Heterozygous	Unknown Significance
F5	rs4525	c.2594A>G		Heterozygous	Unknown Significance
F5	rs6032	c.2773A>G		Heterozygous	Unknown Significance
F5		c.237A>G		Heterozygous	Likely Benign
F5	rs6022	c.552G>T		Heterozygous	Likely Benign
F5	rs6016	c.2208C>T		Heterozygous	Likely Benign
F5	rs6017	c.2235T>C		Heterozygous	Likely Benign
F5	rs6021	c.2301A>G		Heterozygous	Likely Benign
F5	rs1800594	c.3804T>C		Heterozygous	Likely Benign
F5	rs9287090	c.3948C>T		Heterozygous	Likely Benign
F5	rs9332607	c.4095C>T		Heterozygous	Likely Benign
F5	rs6030	c.5290A>G		Heterozygous	Likely Benign

Illumina Clinical Services Laboratory PG0000644-BLD

MOLECULAR DIAGNOSTICS REPORT

Gene	dbSNP ID	cDNA	AA	Zygosity	Intepretation
F5		c.*1601C>T		Heterozygous	Likely Benign
F5	rs6029	c.405G>A		Heterozygous	Benign
F5	rs6025	c.1601A>G		Homozygous	Benign
F5		c.5419+12A>G		Homozygous	Benign
F5		c.*1115C>T		Heterozygous	Benign
F5		c.*2328G>C		Homozygous	Benign
FAH		c.82-13G>A		Homozygous	Benign
FANCA	rs1800282	c.17T>A	p.Val6Asp	Heterozygous	Unknown Significance
FANCA		c.710-12A>G		Homozygous	Benign
FANCA	rs2239359	c.1501G>A	p.Gly501Ser	Homozygous	Benign
FANCC		c.584A>T	p.Asp195Val	Heterozygous	Likely Benign
FANCD2	rs12330369	c.1401G>A	p.Thr467Thr	Heterozygous	Unknown Significance
FANCD2	rs12330599	c.1413+14T>C		Heterozygous	Unknown Significance
FANCD2		c.*550C>T		Heterozygous	Likely Benign
FANCE	rs4713867	c.387A>C	p.Pro129Pro	Heterozygous	Benign
FANCF		c.*207C>T		Homozygous	Benign
FANCF		c.*632G>A		Homozygous	Benign
FANCF		c.*819C>T		Homozygous	Benign
FANCI	rs7183618	c.2547G>A	p.Lys849Lys	Homozygous	Benign
FGD4		c.993+8G>A		Heterozygous	Unknown Significance
FGD4		c.*4991A>G		Homozygous	Benign
FIG4		c.1948+3A>G		Homozygous	Likely Benign
FKTN	rs34787999	c.608G>A	p.Arg203Gln	Heterozygous	Unknown Significance
FKTN		c.-158G>C		Heterozygous	Likely Benign
FKTN	rs17309806	c.1026C>A	p.Leu342Leu	Heterozygous	Likely Benign
G6PD		c.1311C>T	p.Tyr437Tyr	Homozygous	Likely Benign
G6PD		c.1365-13T>C		Homozygous	Likely Benign
GAA	rs1800301	c.642C>T	p.Ser214Ser	Heterozygous	Likely Benign
GAA	rs1800310	c.2133A>G	p.Thr711Thr	Homozygous	Likely Benign
GAA		c.*223C>T		Homozygous	Likely Benign
GAA	rs1800300	c.324T>C	p.Cys108Cys	Homozygous	Benign
GAA		c.547-4C>G		Homozygous	Benign
GAA	rs1042393	c.596A>G	p.His199Arg	Homozygous	Benign
GAA	rs1042395	c.668G>A	p.Arg223His	Homozygous	Benign
GAA		c.955+12G>A		Homozygous	Benign
GAA	rs1800304	c.1203G>A	p.Gln401Gln	Homozygous	Benign
GAA	rs1126690	c.2338G>A	p.Val780Ile	Homozygous	Benign
GAA	rs1042397	c.2553G>A	p.Gly851Gly	Homozygous	Benign
GALC	rs11552556	c.330C>T	p.Asp110Asp	Heterozygous	Unknown Significance
GALC	rs398076	c.1350C>T	p.Ser450Ser	Heterozygous	Likely Benign
GALC		c.*626C>T		Homozygous	Likely Benign
GALC		c.*723G>A		Homozygous	Likely Benign
GALC		c.*1453A>G		Homozygous	Likely Benign

Illumina Clinical Services Laboratory PG0000644-BLD

MOLECULAR DIAGNOSTICS REPORT

Gene	dbSNP ID	cDNA	AA	Zygosity	Intepretation
GALC	rs367327	c.1620A>G	p.Thr540Thr	Homozygous	Benign
GALC	rs398607	c.1685T>C	p.Ile562Thr	Homozygous	Benign
GALC	rs421466	c.1698A>T	p.Val566Val	Homozygous	Benign
GALC		c.1834+5C>G		Homozygous	Benign
GALC	rs421262	c.1921A>G	p.Thr641Ala	Homozygous	Benign
GALC		c.*627A>G		Homozygous	Benign
GALC		c.*989G>A		Homozygous	Benign
GALNS	rs117053987	c.723C>T	p.Ala241Ala	Heterozygous	Unknown Significance
GARS		c.2095-6C>T		Homozygous	Likely Benign
GARS	rs1049402	c.124C>G	p.Pro42Ala	Homozygous	Benign
GATM		c.1252T>C	p.Leu418Leu	Heterozygous	Benign
GATM		c.*940C>T		Heterozygous	Benign
GCDH		c.*288G>T		Heterozygous	Unknown Significance
GCDH		c.*165A>G		Heterozygous	Benign
GCK		c.*332G>A		Heterozygous	Likely Benign
GDAP1	rs11554166	c.507T>G	p.Ser169Ser	Heterozygous	Likely Benign
GDAP1		c.*1855A>G		Heterozygous	Likely Benign
GDAP1		c.*2214A>G		Heterozygous	Likely Benign
GDAP1	rs7828201	c.102G>C	p.Ser34Ser	Homozygous	Benign
GDAP1		c.*797A>G		Homozygous	Benign
GJB2		c.*84T>C		Heterozygous	Benign
GJB2		c.*1067G>T		Heterozygous	Benign
GJB2		c.*1152G>A		Heterozygous	Benign
GJB2		c.*1277T>C		Homozygous	Benign
GJB3		c.*43C>A		Heterozygous	Unknown Significance
GJB3		c.*493C>T		Heterozygous	Unknown Significance
GJB3		c.*53G>A		Heterozygous	Likely Benign
GJB6		c.*337G>T		Heterozygous	Likely Benign
GLB1		c.458-11T>C		Heterozygous	Unknown Significance
GLB1		c.1233+8T>C		Heterozygous	Unknown Significance
GLB1	rs7637099	c.29C>T		Heterozygous	Benign
GLB1	rs7614776	c.34T>C		Heterozygous	Benign
GLB1	rs4302331	c.1561T>C		Homozygous	Benign
GM2A	rs61740602	c.458T>C	p.Val153Ala	Heterozygous	Unknown Significance
GM2A		c.*227A>G		Heterozygous	Likely Benign
GM2A	rs153477	c.175A>G	p.Ile59Val	Heterozygous	Benign
GM2A	rs153478	c.205A>G	p.Met69Val	Heterozygous	Benign
GM2A		c.*944T>C		Heterozygous	Benign
GNPTAB	rs10778148	c.1932A>G	p.Thr644Thr	Heterozygous	Benign
GNPTAB		c.3135+5T>C		Heterozygous	Benign
GNS	rs1147096	c.198G>A	p.Pro66Pro	Homozygous	Benign
GNS		c.*380C>T		Homozygous	Benign

MOLECULAR DIAGNOSTICS REPORT

Gene	dbSNP ID	cDNA	AA	Zygosity	Intepretation
GNS		c.*1353G>A		Homozygous	Benign
GNS		c.*2361C>G		Homozygous	Benign
GPSM2		c.-258C>T		Heterozygous	Unknown Significance
GPSM2		c.380G>A	p.Arg127Gln	Heterozygous	Unknown Significance
GPSM2		c.*211G>A		Heterozygous	Likely Benign
GPSM2		c.*455C>T		Heterozygous	Likely Benign
GRN		c.264+7G>A		Heterozygous	Unknown Significance
GRXCR1	rs78136490	c.25G>A	p.Glu9Lys	Heterozygous	Unknown Significance
GRXCR1		c.627+8A>C		Heterozygous	Benign
GUCA1B		c.*1318G>A		Homozygous	Likely Benign
GUCA1B		c.-17T>C		Homozygous	Benign
GUCA1B	rs3749921	c.171T>C	p.Tyr57Tyr	Homozygous	Benign
HADHA		c.*202G>A		Homozygous	Benign
HADHB		c.-201G>A		Homozygous	Likely Benign
HADHB		c.*136G>C		Homozygous	Benign
HBB	rs713040	c.9T>C	p.His3His	Homozygous	Benign
HEXA	rs1800431	c.1306A>G	p.Ile436Val	Homozygous	Benign
HEXA	rs4777502	c.1518A>G	p.Glu506Glu	Homozygous	Benign
HEXA		c.*515G>A		Homozygous	Benign
HEXA		c.*589T>G		Homozygous	Benign
HEXB	rs820878	c.185T>C		Homozygous	Benign
HEXB	rs11556045	c.362A>G		Heterozygous	Benign
HGSNAT		c.*1780C>T		Heterozygous	Unknown Significance
HGSNAT	rs1126058	c.1749T>C	p.Tyr583Tyr	Homozygous	Benign
HGSNAT		c.*1801T>C		Homozygous	Benign
HNF1A	rs1169305	c.1720A>G		Homozygous	Benign
HPD		c.97A>G		Homozygous	Likely Benign
HPD		c.-55G>A		Homozygous	Benign
HSPB8		c.*684G>A		Heterozygous	Unknown Significance
HSPB8		c.*644A>G		Heterozygous	Likely Benign
IDUA	rs3755954	c.352C>T	p.Leu118Leu	Heterozygous	Likely Benign
IGHMBP2		c.-2C>T		Heterozygous	Unknown Significance
IGHMBP2		c.*665C>T		Heterozygous	Unknown Significance
IGHMBP2		c.*681T>C		Heterozygous	Unknown Significance
IGHMBP2	rs11228413	c.1554C>T	p.Val518Val	Heterozygous	Likely Benign
IGHMBP2	rs622082	c.2011A>G	p.Thr671Ala	Heterozygous	Likely Benign
IGHMBP2	rs546382	c.2316C>T	p.Ser772Ser	Heterozygous	Likely Benign
IGHMBP2	rs560096	c.602T>C	p.Leu201Ser	Homozygous	Benign
IGHMBP2	rs10896380	c.823A>G	p.Ile275Val	Heterozygous	Benign
IGHMBP2	rs2236654	c.2080C>T	p.Arg694Trp	Heterozygous	Benign
IGHMBP2		c.*255T>C		Homozygous	Benign

MOLECULAR DIAGNOSTICS REPORT

Gene	dbSNP ID	cDNA	AA	Zygosity	Intepretation
IKBKAP	rs2230792	c.2294G>A	p.Gly765Glu	Heterozygous	Unknown Significance
IKBKAP		c.3214T>A	p.Cys1072Ser	Heterozygous	Unknown Significance
IKBKAP		c.3473C>T	p.Pro1158Leu	Heterozygous	Unknown Significance
IKBKAP	rs2230793	c.2446A>C	p.Ile816Leu	Heterozygous	Likely Benign
IKBKAP	rs1063110	c.3069G>C	p.Leu1023Leu	Heterozygous	Likely Benign
IKBKAP		c.*1351C>A		Heterozygous	Likely Benign
IVD		c.243+14T>C		Heterozygous	Benign
IVD		c.*808C>G		Heterozygous	Benign
IVD		c.*1471G>A		Heterozygous	Benign
IVD		c.*1577C>G		Heterozygous	Benign
IVD		c.*1610C>T		Heterozygous	Benign
IVD		c.*2844T>C		Heterozygous	Benign
IYD		c.*2742G>A		Heterozygous	Unknown Significance
IYD		c.*3655G>A		Heterozygous	Unknown Significance
IYD		c.*4035G>A		Heterozygous	Unknown Significance
IYD		c.*5218T>C		Heterozygous	Unknown Significance
IYD		c.*5333C>A		Heterozygous	Unknown Significance
IYD		c.*337C>T		Homozygous	Likely Benign
IYD		c.*1121C>G		Heterozygous	Likely Benign
IYD		c.*2072A>G		Heterozygous	Likely Benign
IYD		c.*2509C>T		Heterozygous	Likely Benign
IYD		c.*2815G>A		Heterozygous	Likely Benign
IYD		c.*4243A>G		Heterozygous	Likely Benign
IYD		c.*4295G>A		Heterozygous	Likely Benign
IYD		c.*4439T>C		Heterozygous	Likely Benign
IYD		c.*4691A>C		Homozygous	Likely Benign
IYD		c.*1066C>T		Homozygous	Benign
IYD		c.*1198T>G		Homozygous	Benign
IYD		c.*1535T>C		Homozygous	Benign
IYD		c.*1615G>A		Homozygous	Benign
IYD		c.*1616T>A		Homozygous	Benign
IYD		c.*1856G>A		Heterozygous	Benign
IYD		c.*2028T>C		Heterozygous	Benign
IYD		c.*2814T>C		Homozygous	Benign
IYD		c.*3995A>C		Homozygous	Benign
JAG1	rs45534738	c.*756A>G		Heterozygous	Unknown Significance
JAG1	rs1131695	c.765C>T	p.Tyr255Tyr	Heterozygous	Likely Benign
JAG1		c.*1572A>G		Homozygous	Likely Benign
JAG1	rs1051419	c.3417T>C	p.Tyr1139Tyr	Homozygous	Benign
JUP		c.-88A>G		Heterozygous	Benign
JUP	rs7405731	c.213T>C	p.Asp71Asp	Homozygous	Benign

Illumina Clinical Services Laboratory PG0000644-BLD

MOLECULAR DIAGNOSTICS REPORT

Gene	dbSNP ID	cDNA	AA	Zygosity	Intepretation
JUP	rs1126821	c.2089A>T	p.Met697Leu	Heterozygous	Benign
JUP		c.*225T>C		Heterozygous	Benign
KCNE1		c.*124A>G		Homozygous	Likely Benign
KCNE1		c.*456C>T		Homozygous	Likely Benign
KCNE1		c.*2480A>G		Homozygous	Likely Benign
KCNE1	rs17846179	c.112A>G	p.Ser38Gly	Homozygous	Benign
KCNE1		c.*1219A>G		Homozygous	Benign
KCNE1		c.*2007T>C		Homozygous	Benign
KCNE1		c.*2529C>T		Homozygous	Benign
KCNE3		c.*1977A>C		Heterozygous	Likely Benign
KCNE3		c.*2019C>T		Heterozygous	Benign
KCNJ2		c.*1794C>A		Heterozygous	Unknown Significance
KCNQ1		c.1394-14C>T		Heterozygous	Unknown Significance
KCNQ1	rs11601907	c.1986C>T	p.Tyr662Tyr	Heterozygous	Likely Benign
KIF1B	rs12125492	c.4161A>G	p.Pro1387Pro	Heterozygous	Unknown Significance
KIF1B		c.*1133C>T		Heterozygous	Unknown Significance
KIF1B		c.*1159G>A		Heterozygous	Unknown Significance
KIF1B		c.*2042C>T		Heterozygous	Benign
KLHL7	rs15775	c.352C>T	p.Leu118Leu	Homozygous	Benign
LAMP2		c.*4579A>G		Homozygous	Benign
LDB3		c.-114T>C		Heterozygous	Benign
LDLR		c.*1217C>G		Homozygous	Unknown Significance
LDLR	rs688	c.1773C>T	p.Asn591Asn	Homozygous	Likely Benign
LDLR	rs5925	c.1959T>C	p.Val653Val	Homozygous	Likely Benign
LDLR		c.*1268C>T		Homozygous	Likely Benign
LDLR		c.*1743C>T		Homozygous	Likely Benign
LDLR		c.1060+7T>C		Homozygous	Benign
LDLR		c.1060+10G>C		Homozygous	Benign
LDLR	rs5930	c.1413A>G	p.Arg471Arg	Homozygous	Benign
LDLR	rs5927	c.2232A>G	p.Arg744Arg	Homozygous	Benign
LDLR		c.*315G>C		Homozygous	Benign
LDLR		c.*666T>C		Homozygous	Benign
LDLR		c.*1262T>C		Homozygous	Benign
LIPA	rs1051338	c.46A>C	p.Thr16Pro	Heterozygous	Unknown Significance
LIPA		c.*909T>A		Heterozygous	Unknown Significance
LITAF		c.*796T>C		Heterozygous	Unknown Significance
LITAF		c.*1251A>G		Heterozygous	Likely Benign
LOXHD1	rs12606417	c.4868A>G	p.Glu1623Gly	Heterozygous	Unknown Significance
LOXHD1		c.5213+13G>A		Heterozygous	Unknown Significance

Illumina Clinical Services Laboratory PG0000644-BLD

MOLECULAR DIAGNOSTICS REPORT

Gene	dbSNP ID	cDNA	AA	Zygosity	Intepretation
LOXHD1		c.6398G>A	p.Arg2133His	Heterozygous	Unknown Significance
LOXHD1	rs1377016	c.6107C>T	p.Ala2036Val	Heterozygous	Likely Benign
LOXHD1	rs1893566	c.3463A>G	p.Arg1155Gly	Homozygous	Benign
LRRK2		c.2857T>C		Heterozygous	Likely Benign
LRRK2	rs2256408	c.149G>A		Homozygous	Benign
LRRK2	rs10878245	c.457T>C		Homozygous	Benign
MANBA	rs2866413	c.2102C>T	p.Thr701Met	Heterozygous	Benign
MANBA	rs2272697	c.2368T>C	p.Leu790Leu	Heterozygous	Benign
MANBA		c.*124T>C		Heterozygous	Benign
MANBA		c.*161C>T		Heterozygous	Benign
MANBA		c.*384C>T		Heterozygous	Benign
MANBA		c.*401G>A		Heterozygous	Benign
MANBA		c.*505G>A		Heterozygous	Benign
MAPT		c.-285A>G		Heterozygous	Unknown Significance
MAPT		c.-133C>A		Heterozygous	Unknown Significance
MAPT		c.-13A>G		Heterozygous	Unknown Significance
MAPT		c.307+9A>G		Heterozygous	Unknown Significance
MAPT		c.*26T>C		Heterozygous	Unknown Significance
MAPT		c.*334A>G		Heterozygous	Unknown Significance
MAPT		c.*1067T>C		Heterozygous	Unknown Significance
MAPT		c.*1101A>G		Heterozygous	Unknown Significance
MAPT		c.*1152G>C		Heterozygous	Unknown Significance
MAPT		c.*1328A>C		Heterozygous	Unknown Significance
MAPT		c.*1396T>C		Heterozygous	Unknown Significance
MAPT		c.*1759T>C		Heterozygous	Unknown Significance
MAPT		c.*2079C>T		Heterozygous	Unknown Significance
MAPT		c.*2288T>C		Heterozygous	Unknown Significance
MAPT		c.*2289G>A		Heterozygous	Unknown Significance
MAPT		c.*2806A>C		Heterozygous	Unknown Significance
MAPT		c.*2972T>C		Heterozygous	Unknown Significance
MAPT	rs116444268	c.765T>C	p.Asn255Asn	Heterozygous	Likely Benign
MAPT	rs114553892	c.681A>G	p.Ala227Ala	Heterozygous	Benign
MAPT		c.*3858A>G		Heterozygous	Benign
MARVELD2		c.1147-9T>G		Homozygous	Likely Benign

MOLECULAR DIAGNOSTICS REPORT

Gene	dbSNP ID	cDNA	AA	Zygosity	Intepretation
MAT1A		c.*67C>T		Heterozygous	Unknown Significance
MAT1A	rs77083841	c.*610G>C		Heterozygous	Unknown Significance
MAT1A		c.*1206G>A		Heterozygous	Unknown Significance
MAT1A	rs1143694	c.426T>C	p.Ala142Ala	Homozygous	Benign
MAT1A	rs10788546	c.870A>G	p.Val290Val	Homozygous	Benign
MAT1A	rs10887711	c.882T>C	p.Ala294Ala	Homozygous	Benign
MAT1A		c.1085+14C>T		Heterozygous	Benign
MAT1A	rs2993763	c.1131C>T	p.Tyr377Tyr	Heterozygous	Benign
MAT1A		c.*1297T>C		Heterozygous	Benign
MCCC1	rs7622479	c.396C>T	p.Leu132Leu	Homozygous	Benign
MCCC1	rs2270968	c.1391A>C	p.His464Pro	Homozygous	Benign
MCCC2		c.*593G>T		Heterozygous	Unknown Significance
MCCC2		c.-117A>G		Homozygous	Benign
MCCC2	rs10064079	c.1368A>G	p.Ala456Ala	Homozygous	Benign
MCCC2		c.*247C>T		Homozygous	Benign
MCCC2		c.*1243G>C		Homozygous	Benign
MCEE	rs11541017	c.227C>T	p.Ala76Val	Homozygous	Likely Benign
MEFV	rs1231122	c.1764G>A	p.Pro588Pro	Heterozygous	Likely Benign
MEFV	rs224225	c.306T>C	p.Asp102Asp	Heterozygous	Benign
MEFV	rs224224	c.414A>G	p.Gly138Gly	Heterozygous	Benign
MEFV	rs224223	c.495C>A	p.Ala165Ala	Heterozygous	Benign
MEFV	rs224213	c.942C>T	p.Arg314Arg	Heterozygous	Benign
MEFV	rs224208	c.1422G>A	p.Glu474Glu	Heterozygous	Benign
MEFV	rs224207	c.1428A>G	p.Gln476Gln	Heterozygous	Benign
MEFV	rs224206	c.1530T>C	p.Asp510Asp	Heterozygous	Benign
MEFV		c.*245G>A		Heterozygous	Benign
MEFV		c.*267G>A		Heterozygous	Benign
MEFV		c.*1056G>T		Heterozygous	Benign
MERTK		c.757+13T>C		Heterozygous	Likely Benign
MERTK		c.2080-11C>A		Heterozygous	Likely Benign
MERTK	rs7604639	c.1397G>A	p.Arg466Lys	Heterozygous	Benign
MERTK	rs2230515	c.1552A>G	p.Ile518Val	Heterozygous	Benign
MERTK	rs1131244	c.1881A>G	p.Ser627Ser	Heterozygous	Benign
MFN2		c.*58A>G		Homozygous	Benign
MFN2		c.*896G>C		Homozygous	Benign
MLH1		c.-93G>A		Heterozygous	Likely Benign
MLH1		c.655A>G	p.Ile219Val	Heterozygous	Likely Benign
MLH3		c.2531C>T	p.Pro844Leu	Heterozygous	Unknown Significance
MLH3		c.*957C>A		Heterozygous	Likely Benign
MLH3		c.*958G>T		Heterozygous	Likely Benign
MLH3		c.*2417G>A		Heterozygous	Likely Benign
MLH3		c.*3148A>G		Heterozygous	Likely Benign
MLH3	rs175081	c.2476A>G	p.Asn826Asp	Homozygous	Benign
MLH3	rs13712	c.4335A>G	p.Gln1445Gln	Heterozygous	Benign

MOLECULAR DIAGNOSTICS REPORT

Gene	dbSNP ID	cDNA	AA	Zygosity	Intepretation
MMAA	rs11721553	c.747G>A	p.Ser249Ser	Heterozygous	Unknown Significance
MMAA		c.*2608T>C		Heterozygous	Benign
MMAA		c.*2768A>T		Heterozygous	Benign
MMAB		c.56G>A	p.Arg19His	Heterozygous	Unknown Significance
MMAB		c.*380A>T		Heterozygous	Unknown Significance
MMAB	rs143292900	c.*1067C>T		Heterozygous	Unknown Significance
MMAB		c.*1409G>A		Heterozygous	Unknown Significance
MMAB		c.57C>A	p.Arg19Arg	Heterozygous	Likely Benign
MMAB		c.*625G>C		Heterozygous	Likely Benign
MMAB		c.*891C>A		Heterozygous	Likely Benign
MMAB		c.*3148G>A		Heterozygous	Likely Benign
MMAB	rs9593	c.716T>A	p.Met239Lys	Heterozygous	Benign
MMAB		c.*857G>C		Heterozygous	Benign
MMAB		c.*1230G>T		Heterozygous	Benign
MMADHC	rs11545261	c.453G>A	p.Gln151Gln	Heterozygous	Benign
MMADHC		c.*89T>C		Homozygous	Benign
MMADHC		c.*126A>G		Heterozygous	Benign
MPI		c.345+15G>A		Heterozygous	Likely Benign
MPI		c.670+9A>G		Heterozygous	Likely Benign
MPI	rs1130741	c.1131A>G	p.Val377Val	Heterozygous	Benign
MPZ		c.*761A>G		Heterozygous	Unknown Significance
MSH6		c.1186C>G	p.Leu396Val	Heterozygous	Unknown Significance
MSH6	rs1800935	c.540T>C	p.Asp180Asp	Heterozygous	Likely Benign
MSH6	rs1800937	c.642C>T	p.Tyr214Tyr	Heterozygous	Likely Benign
MTMR2	rs3824874	c.8A>C	p.Lys3Thr	Heterozygous	Likely Benign
MTMR2	rs566204	c.1131C>T	p.Thr377Thr	Heterozygous	Likely Benign
MTMR2		c.*549G>A		Heterozygous	Likely Benign
MTMR2		c.*1419G>A		Heterozygous	Likely Benign
MTMR2		c.*1568C>T		Heterozygous	Likely Benign
MTMR2		c.*1934T>C		Heterozygous	Likely Benign
MUT		c.-198A>T		Heterozygous	Likely Benign
MUT	rs1141321	c.1595G>A	p.Arg532His	Heterozygous	Likely Benign
MUT	rs2229384	c.636G>A	p.Lys212Lys	Homozygous	Benign
MUT		c.1495G>A	p.Ala499Thr	Heterozygous	Benign
MUT	rs8589	c.2011A>G	p.Ile671Val	Homozygous	Benign
MYH14		c.3557+9C>T		Heterozygous	Unknown Significance
MYH14		c.1090+13C>T		Heterozygous	Likely Benign
MYH14		c.2232-14C>T		Homozygous	Likely Benign
MYH14	rs4801822	c.657G>A	p.Ala219Ala	Homozygous	Benign
MYH14	rs1651553	c.2127A>G	p.Pro709Pro	Homozygous	Benign
MYH14	rs3745504	c.2895G>A	p.Leu965Leu	Homozygous	Benign

Illumina Clinical Services Laboratory PG0000644-BLD

MOLECULAR DIAGNOSTICS REPORT

Gene	dbSNP ID	cDNA	AA	Zygosity	Intepretation
MYH14		c.2910+11T>C		Homozygous	Benign
MYH14	rs3745509	c.5307G>A	p.Ser1769Ser	Heterozygous	Benign
MYH14		c.5555+12C>T		Heterozygous	Benign
MYH14		c.*216T>G		Heterozygous	Benign
MYH7	rs735711	c.1095G>A	p.Lys365Lys	Heterozygous	Unknown Significance
MYH7	rs7157716	c.2967T>C	p.Ile989Ile	Heterozygous	Likely Benign
MYH7	rs2069540	c.189C>T	p.Thr63Thr	Heterozygous	Benign
MYH9		c.1554+7A>G		Homozygous	Likely Benign
MYH9		c.*596G>A		Heterozygous	Likely Benign
MYH9		c.1728+10G>A		Homozygous	Benign
MYH9	rs710181	c.3429T>G	p.Ala1143Ala	Homozygous	Benign
MYO15A		c.3140C>G	p.Pro1047Arg	Heterozygous	Unknown Significance
MYO15A		c.-219-14T>C		Heterozygous	Benign
MYO15A		c.-76A>C		Heterozygous	Benign
MYO15A	rs2955365	c.1783G>A	p.Ala595Thr	Heterozygous	Benign
MYO15A	rs2955366	c.1899A>G	p.Pro633Pro	Heterozygous	Benign
MYO15A	rs2955367	c.2152T>G	p.Trp718Gly	Heterozygous	Benign
MYO15A	rs2955379	c.7185T>C	p.Phe2395Phe	Homozygous	Benign
MYO3A		c.624C>T	p.Asp208Asp	Heterozygous	Unknown Significance
MYO3A		c.660C>T	p.Ala220Ala	Heterozygous	Unknown Significance
MYO3A	rs3824700	c.956G>A	p.Arg319His	Heterozygous	Likely Benign
MYO3A		c.1053+11C>T		Heterozygous	Likely Benign
MYO3A		c.1053+12A>G		Heterozygous	Likely Benign
MYO3A	rs35379457	c.1104C>T	p.Tyr368Tyr	Heterozygous	Likely Benign
MYO3A		c.1170+7C>T		Heterozygous	Likely Benign
MYO3A	rs3824699	c.1042A>G	p.Ile348Val	Heterozygous	Benign
MYO3A	rs3817420	c.1105G>A	p.Val369Ile	Heterozygous	Benign
MYO6		c.553+11T>C		Heterozygous	Unknown Significance
MYO6		c.1722C>T	p.Asp574Asp	Heterozygous	Unknown Significance
MYO6		c.*459A>G		Heterozygous	Unknown Significance
MYO6		c.*2027T>C		Heterozygous	Likely Benign
MYO6		c.*4283A>G		Heterozygous	Likely Benign
MYO6		c.*3835C>T		Homozygous	Benign
MYO7A	rs948962	c.5860C>A	p.Leu1954Ile	Homozygous	Likely Benign
MYO7A		c.6318G>A	p.Lys2106Lys	Heterozygous	Likely Benign
MYO7A	rs762667	c.783T>C	p.Gly261Gly	Heterozygous	Benign
MYO7A		c.3924+12C>T		Homozygous	Benign
MYO7A	rs7927472	c.4755C>T	p.Ser1585Ser	Homozygous	Benign
MYO7A	rs2276288	c.4996A>T	p.Ser1666Cys	Homozygous	Benign
MYO7A	rs2276293	c.5715A>G	p.Lys1905Lys	Homozygous	Benign
MYO7A		c.5743-12T>C		Homozygous	Benign
MYO7A		c.5857-7A>T		Homozygous	Benign

Illumina Clinical Services Laboratory PG0000644-BLD

MOLECULAR DIAGNOSTICS REPORT

Gene	dbSNP ID	cDNA	AA	Zygosity	Intepretation
MYOT	rs41431944	c.220A>C		Homozygous	Benign
MYOZ2		c.*1248G>A		Heterozygous	Likely Benign
NAGA		c.*1103T>C		Heterozygous	Unknown Significance
NAGLU	rs659497	c.423T>C	p.Ser141Ser	Homozygous	Benign
NAGLU	rs86312	c.2209C>G	p.Arg737Gly	Homozygous	Benign
NDRG1		c.64-6T>C		Homozygous	Benign
NEFL		c.*1155T>G		Heterozygous	Unknown Significance
NEFL		c.*235A>T		Homozygous	Benign
NEFL		c.*687G>C		Homozygous	Benign
NEFL		c.*1256G>A		Homozygous	Benign
NF1	rs1801052	c.702G>A	p.Leu234Leu	Heterozygous	Likely Benign
NF1		c.*2201G>A		Heterozygous	Benign
NF1		c.*2265C>G		Heterozygous	Benign
NF1		c.*2829T>C		Heterozygous	Benign
NF2		c.*1792G>A		Homozygous	Unknown Significance
NF2		c.*1904A>G		Homozygous	Unknown Significance
NF2		c.*2235G>A		Homozygous	Unknown Significance
NF2		c.*3274G>A		Homozygous	Unknown Significance
NF2		c.*354T>C		Homozygous	Likely Benign
NF2		c.*1208C>T		Homozygous	Likely Benign
NHLRC1	rs10949483	c.332C>T	p.Pro111Leu	Heterozygous	Benign
NLRP12		c.*266G>T		Heterozygous	Unknown Significance
NLRP12		c.-12C>T		Homozygous	Likely Benign
NLRP12		c.-140T>A		Homozygous	Benign
NLRP12	rs4806773	c.2394G>A	p.Gln798Gln	Heterozygous	Benign
NLRP12	rs12460528	c.2469C>T	p.Leu823Leu	Heterozygous	Benign
NLRP3		c.-405T>C		Homozygous	Benign
NLRP3	rs3806268	c.732G>A	p.Ala244Ala	Heterozygous	Benign
NLRP3	rs4925543	c.786A>G	p.Arg262Arg	Heterozygous	Benign
NLRP3		c.*230G>C		Heterozygous	Benign
NLRP3		c.*489C>T		Heterozygous	Benign
NPC1	rs1805081	c.644A>G	p.His215Arg	Heterozygous	Unknown Significance
NPC1	rs1788799	c.1926G>C	p.Met642Ile	Heterozygous	Benign
NPC1	rs1805082	c.2572A>G	p.Ile858Val	Heterozygous	Benign
NPC1	rs1140458	c.2793C>T	p.Asn931Asn	Heterozygous	Benign
NRL		c.*221G>A		Homozygous	Likely Benign
OPA3		c.-38A>G		Heterozygous	Unknown Significance
OPA3		c.*638T>A		Heterozygous	Unknown Significance
OPA3	rs3826860	c.231T>C	p.Ala77Ala	Heterozygous	Benign
OPA3		c.*152G>A		Heterozygous	Benign

Illumina Clinical Services Laboratory PG0000644-BLD

MOLECULAR DIAGNOSTICS REPORT

Gene	dbSNP ID	cDNA	AA	Zygosity	Intepretation
OPA3		c.*1950G>A		Heterozygous	Benign
OPA3		c.*3387G>A		Heterozygous	Benign
OPA3		c.*4318T>C		Heterozygous	Benign
OPA3		c.*6790T>C		Homozygous	Benign
OTOF	rs13031859	c.244C>T	p.Arg82Cys	Heterozygous	Likely Benign
OTOF	rs11687696	c.372A>G	p.Thr124Thr	Heterozygous	Likely Benign
OTOF	rs2272069	c.2580C>G	p.Val860Val	Heterozygous	Benign
OTOF	rs4335905	c.2736G>C	p.Leu912Leu	Heterozygous	Benign
PAH		c.-71A>C		Heterozygous	Unknown Significance
PAH	rs1042503	c.735G>A	p.Val245Val	Heterozygous	Likely Benign
PAH	rs772897	c.1155C>G	p.Leu385Leu	Heterozygous	Benign
PAX8		c.*1000T>C		Heterozygous	Unknown Significance
PAX8		c.*2146A>G		Heterozygous	Likely Benign
PAX8		c.*2478C>T		Heterozygous	Likely Benign
PAX8		c.-104C>G		Homozygous	Benign
PAX8		c.*2309A>G		Heterozygous	Benign
PCDH15		c.706-8C>T		Heterozygous	Benign
PCSK9		c.207+15A>G		Homozygous	Benign
PCSK9	rs509504	c.1026A>G	p.Gln342Gln	Homozygous	Benign
PCSK9	rs540796	c.1380A>G	p.Val460Val	Homozygous	Benign
PCSK9	rs562556	c.1420G>A	p.Val474Ile	Homozygous	Benign
PCSK9	rs505151	c.2009G>A	p.Gly670Glu	Homozygous	Benign
PCSK9		c.*571C>T		Homozygous	Benign
PDE6A		c.*2194G>A		Heterozygous	Likely Benign
PDE6A		c.*2336C>T		Heterozygous	Likely Benign
PDE6A		c.*2542G>A		Heterozygous	Likely Benign
PDE6A		c.*608T>C		Heterozygous	Benign
PDE6A		c.*756T>C		Heterozygous	Benign
PDE6A		c.*796A>G		Heterozygous	Benign
PDE6A		c.*1365G>A		Heterozygous	Benign
PDE6A		c.*2009T>C		Heterozygous	Benign
PDE6B		c.655T>C		Heterozygous	Unknown Significance
PDE6B	rs10902758	c.958G>A		Homozygous	Benign
PDE6B		c.*12A>G		Heterozygous	Benign
PHOX2B		c.*1662A>T		Heterozygous	Unknown Significance
PHYH		c.*65G>A		Heterozygous	Likely Benign
PHYH	rs1747682	c.153C>T	p.Asn51Asn	Homozygous	Benign
PINK1		c.*415C>G		Heterozygous	Unknown Significance
PINK1		c.388-7A>G		Heterozygous	Benign
PINK1		c.960-5G>A		Heterozygous	Benign
PINK1		c.*37A>T		Heterozygous	Benign
PINK1		c.*181C>G		Heterozygous	Benign
PINK1		c.*265G>T		Heterozygous	Benign

Illumina Clinical Services Laboratory PG0000644-BLD

MOLECULAR DIAGNOSTICS REPORT

Gene	dbSNP ID	cDNA	AA	Zygosity	Intepretation
PKD2		c.*1237G>A		Heterozygous	Unknown Significance
PKHD1		c.1587T>C	p.Asn529Asn	Heterozygous	Unknown Significance
PKHD1		c.2489A>G	p.Asn830Ser	Heterozygous	Unknown Significance
PKHD1	rs9474143	c.234C>T	p.Asp78Asp	Heterozygous	Likely Benign
PKHD1		c.6854G>A	p.Gly2285Glu	Heterozygous	Likely Benign
PKHD1	rs12210295	c.7587G>A	p.Gly2529Gly	Heterozygous	Likely Benign
PKHD1		c.8302+12T>A		Homozygous	Likely Benign
PKHD1	rs1896976	c.1185T>C	p.Asp395Asp	Homozygous	Benign
PKHD1	rs2435322	c.5608T>G	p.Leu1870Val	Homozygous	Benign
PKHD1		c.7734-4T>C		Heterozygous	Benign
PKHD1	rs9349603	c.7764A>G	p.Leu2588Leu	Heterozygous	Benign
PKHD1	rs4715227	c.11696A>G	p.Gln3899Arg	Heterozygous	Benign
PKHD1	rs9381994	c.12143A>G	p.Gln4048Arg	Heterozygous	Benign
PKHD1		c.*374T>C		Homozygous	Benign
PKHD1		c.*3026A>G		Heterozygous	Benign
PKHD1		c.*3393C>A		Heterozygous	Benign
PKP2		c.*1196G>A		Homozygous	Unknown Significance
PKP2		c.*812C>T		Homozygous	Likely Benign
PKP2		c.*251C>G		Homozygous	Benign
PKP2		c.*944C>A		Homozygous	Benign
PKP2		c.*1431G>A		Homozygous	Benign
PLN		c.*397T>G		Heterozygous	Likely Benign
PLOD1	rs7551175	c.295G>A	p.Ala99Thr	Heterozygous	Likely Benign
PMP22		c.*577T>C		Heterozygous	Unknown Significance
PMP22		c.*59A>C		Heterozygous	Benign
POMGNT1	rs2292487	c.681A>G		Heterozygous	Likely Benign
POMGNT1	rs6659553	c.1867A>G		Homozygous	Benign
POMT1		c.751C>T		Heterozygous	Unknown Significance
POMT1		c.*421G>A		Heterozygous	Unknown Significance
POMT1	rs2296949	c.752A>G		Homozygous	Benign
POMT1	rs10901065	c.942T>C		Homozygous	Benign
POMT1	rs3739494	c.1113T>C		Homozygous	Benign
POMT1		c.2069+13C>T		Homozygous	Benign
POMT1		c.*41T>C		Homozygous	Benign
POMT1		c.*226T>C		Homozygous	Benign
POMT1		c.*278T>C		Homozygous	Benign
POMT1		c.*285A>G		Homozygous	Benign
POMT1		c.*348C>T		Homozygous	Benign
POMT1		c.*453T>C		Heterozygous	Benign
POU3F4	rs5921978	c.708A>G	p.Glu236Glu	Homozygous	Benign
POU3F4	rs5921979	c.710G>C	p.Gly237Ala	Homozygous	Benign
PPT1		c.-83A>G		Homozygous	Benign

Illumina Clinical Services Laboratory PG0000644-BLD

MOLECULAR DIAGNOSTICS REPORT

Gene	dbSNP ID	cDNA	AA	Zygosity	Intepretation
PPT1		c.*285T>G		Heterozygous	Benign
PPT1		c.*505C>G		Heterozygous	Benign
PPT1		c.*657G>A		Heterozygous	Benign
PRKAG2		c.*112A>G		Heterozygous	Benign
PRKAG2		c.*522G>T		Heterozygous	Benign
PRKAG2		c.*1061G>A		Heterozygous	Benign
PROC		c.-50A>T		Heterozygous	Likely Benign
PROC	rs5937	c.768T>C	p.Asp256Asp	Heterozygous	Likely Benign
PROC	rs5936	c.423G>T	p.Ser141Ser	Homozygous	Benign
PROS1	rs6123	c.2001A>G	p.Pro667Pro	Heterozygous	Likely Benign
PROS1		c.*520A>C		Heterozygous	Benign
PRPF31		c.-9+14A>G		Heterozygous	Likely Benign
PRPF31		c.1147-9T>C		Homozygous	Benign
PRPF8		c.993-7A>G		Heterozygous	Unknown Significance
PRPF8		c.4639-13G>A		Heterozygous	Unknown Significance
PRPF8		c.6294G>A	p.Lys2098Lys	Heterozygous	Unknown Significance
PRPF8	rs11559305	c.637T>C	p.Leu213Leu	Heterozygous	Likely Benign
PRPF8	rs7503397	c.891T>C	p.Asn297Asn	Homozygous	Likely Benign
PRPF8	rs33965342	c.2847G>A	p.Pro949Pro	Homozygous	Likely Benign
PRPF8		c.3774+6G>A		Homozygous	Likely Benign
PRPF8		c.6588T>C	p.His2196His	Heterozygous	Likely Benign
PRPH2		c.318T>C	p.Val106Val	Heterozygous	Benign
PRPH2	rs425876	c.929G>A	p.Arg310Lys	Heterozygous	Benign
PSEN1		c.*672G>A		Heterozygous	Unknown Significance
PSEN1	rs362387	c.*1381G>A		Heterozygous	Unknown Significance
PSEN1		c.*3722C>T		Heterozygous	Likely Benign
PSEN1		c.*1147C>T		Homozygous	Benign
PSEN2	rs11405	c.69T>C	p.Ala23Ala	Homozygous	Benign
PSEN2	rs6759	c.129C>T	p.Asn43Asn	Homozygous	Benign
PSEN2	rs1046240	c.261C>T	p.His87His	Homozygous	Benign
RAB7A		c.219C>T	p.Leu73Leu	Heterozygous	Unknown Significance
RAD51C		c.859A>G	p.Thr287Ala	Heterozygous	Unknown Significance
RAG1		c.-65A>G		Heterozygous	Unknown Significance
RAG1		c.2880A>G		Heterozygous	Unknown Significance
RAG1		c.*1083C>T		Heterozygous	Unknown Significance
RAG1		c.*2246T>C		Heterozygous	Unknown Significance
RAG1	rs3740955	c.746A>G		Heterozygous	Benign
RAG1		c.*3184G>A		Heterozygous	Benign

Illumina Clinical Services Laboratory PG0000644-BLD

MOLECULAR DIAGNOSTICS REPORT

Gene	dbSNP ID	cDNA	AA	Zygosity	Intepretation
RAG2		c.-137T>C		Heterozygous	Unknown Significance
RAG2		c.*328A>G		Heterozygous	Benign
RBM20		c.*546C>T		Homozygous	Unknown Significance
RBM20		c.*1553G>A		Homozygous	Unknown Significance
RBM20		c.*1104G>A		Homozygous	Likely Benign
RBM20	rs1417635	c.2303G>C	p.Trp768Ser	Homozygous	Benign
RBM20		c.3452-9G>C		Homozygous	Benign
RBM20	rs942077	c.3667G>C	p.Glu1223Gln	Homozygous	Benign
RDH12		c.482G>A	p.Arg161Gln	Heterozygous	Unknown Significance
RET		c.337+9G>A		Heterozygous	Unknown Significance
RET		c.2071G>A	p.Gly691Ser	Heterozygous	Unknown Significance
RET		c.*388G>A		Heterozygous	Unknown Significance
RET		c.2712C>G	p.Ser904Ser	Heterozygous	Likely Benign
RET	rs1800858	c.135A>G	p.Ala45Ala	Heterozygous	Benign
RET	rs1800860	c.1296A>G	p.Ala432Ala	Heterozygous	Benign
RET	rs1800861	c.2307G>T	p.Leu769Leu	Heterozygous	Benign
RET		c.*1506G>A		Heterozygous	Benign
RGR		c.19C>T	p.Leu7Leu	Heterozygous	Unknown Significance
RGR		c.*65A>G		Heterozygous	Likely Benign
RGR	rs1042454	c.459C>T	p.Tyr153Tyr	Heterozygous	Benign
RHO		c.*925T>C		Heterozygous	Unknown Significance
RHO		c.-26A>G		Heterozygous	Likely Benign
RHO		c.*912A>G		Heterozygous	Likely Benign
ROM1		c.-22T>C		Homozygous	Benign
ROM1	rs1799959	c.353G>C		Homozygous	Benign
RP1	rs444772	c.2615G>A	p.Arg872His	Heterozygous	Likely Benign
RP1	rs2293869	c.2953A>T	p.Asn985Tyr	Heterozygous	Likely Benign
RP1	rs446227	c.5008G>A	p.Ala1670Thr	Heterozygous	Likely Benign
RP1	rs414352	c.5071T>C	p.Ser1691Pro	Heterozygous	Likely Benign
RP1	rs441800	c.5175A>G	p.Gln1725Gln	Heterozygous	Likely Benign
RP1	rs61739567	c.6098G>A	p.Cys2033Tyr	Heterozygous	Likely Benign
RP2		c.*828G>A		Homozygous	Benign
RYR2	rs17686573	c.1863C>T	p.His621His	Heterozygous	Unknown Significance
RYR2		c.8873A>G	p.Gln2958Arg	Homozygous	Unknown Significance
RYR2		c.11963-11T>C		Heterozygous	Likely Benign
RYR2		c.677-11T>A		Heterozygous	Benign
RYR2	rs3765097	c.1359C>T	p.Ser453Ser	Homozygous	Benign
RYR2		c.1612+14T>C		Homozygous	Benign
RYR2	rs2253273	c.2973A>G	p.Ser991Ser	Homozygous	Benign

Illumina Clinical Services Laboratory PG0000644-BLD

MOLECULAR DIAGNOSTICS REPORT

Gene	dbSNP ID	cDNA	AA	Zygosity	Intepretation
RYR2	rs707189	c.6906T>C	p.Leu2302Leu	Homozygous	Benign
RYR2	rs684923	c.7806C>T	p.His2602His	Homozygous	Benign
RYR2	rs2797436	c.9318T>G	p.Ser3106Ser	Homozygous	Benign
RYR2	rs2797441	c.10503C>T	p.Thr3501Thr	Homozygous	Benign
RYR2	rs2685301	c.10776C>T	p.Ser3592Ser	Homozygous	Benign
RYR2		c.13783-6A>G		Homozygous	Benign
RYR2		c.13913+12A>C		Homozygous	Benign
SAG	rs72976383	c.201C>T	p.Cys67Cys	Heterozygous	Unknown Significance
SAG		c.1208T>C	p.Val403Ala	Heterozygous	Unknown Significance
SBF2		c.3646C>G	p.Gln1216Glu	Heterozygous	Unknown Significance
SBF2		c.*514C>T		Homozygous	Likely Benign
SBF2		c.*1515G>A		Homozygous	Likely Benign
SBF2		c.4571-6C>T		Homozygous	Benign
SBF2		c.*1364C>T		Homozygous	Benign
SCN1B		c.*42T>C		Heterozygous	Unknown Significance
SCN1B		c.*86A>C		Heterozygous	Unknown Significance
SCN4B		c.*2120A>C		Heterozygous	Unknown Significance
SCN4B		c.*2623A>G		Heterozygous	Unknown Significance
SCN4B		c.*2879T>C		Heterozygous	Unknown Significance
SCN4B		c.*1334G>A		Heterozygous	Likely Benign
SCN4B		c.*785G>A		Heterozygous	Benign
SCN4B		c.*3054T>C		Heterozygous	Benign
SCN5A		c.*1537T>C		Heterozygous	Likely Benign
SCN5A	rs6599230	c.87A>G	p.Ala29Ala	Homozygous	Benign
SCN5A	rs7430407	c.3183A>G	p.Glu1061Glu	Heterozygous	Benign
SCN5A		c.5457T>C	p.Asp1819Asp	Heterozygous	Benign
SCN5A		c.*123A>G		Heterozygous	Benign
SCN5A		c.*962T>A		Heterozygous	Benign
SCN5A		c.*963C>T		Heterozygous	Benign
SERPINA1		c.424C>T	p.Leu142Leu	Heterozygous	Unknown Significance
SERPINA1	rs1303	c.1200A>C	p.Glu400Asp	Homozygous	Unknown Significance
SERPINA1		c.*1067G>A		Homozygous	Unknown Significance
SERPINA1		c.*224G>A		Homozygous	Likely Benign
SERPINA1	rs709932	c.374G>A	p.Arg125His	Heterozygous	Benign
SERPINA1		c.*968T>C		Homozygous	Benign
SERPINA1		c.*1221A>G		Homozygous	Benign
SERPINC1	rs5877	c.981A>G	p.Val327Val	Heterozygous	Benign
SERPINC1	rs5878	c.1011A>G	p.Gln337Gln	Heterozygous	Benign

Illumina Clinical Services Laboratory PG0000644-BLD

MOLECULAR DIAGNOSTICS REPORT

Gene	dbSNP ID	cDNA	AA	Zygosity	Intepretation
SERPINE1		c.*1186C>T		Heterozygous	Unknown Significance
SERPINE1		c.*361T>C		Heterozygous	Likely Benign
SERPINE1		c.*722T>G		Heterozygous	Likely Benign
SETX	rs9411449	c.1077T>C	p.Tyr359Tyr	Homozygous	Benign
SETX	rs1185193	c.3576T>G	p.Asp1192Glu	Homozygous	Benign
SETX	rs543573	c.4156A>G	p.Ile1386Val	Homozygous	Benign
SGCA		c.*6T>C		Homozygous	Benign
SGCB		c.*521A>T		Heterozygous	Likely Benign
SGCB		c.*1566T>C		Heterozygous	Likely Benign
SGCB		c.*1624C>T		Heterozygous	Likely Benign
SGCB		c.*2097T>G		Homozygous	Benign
SGCD		c.-94C>G		Heterozygous	Likely Benign
SGCD		c.*1527C>T		Heterozygous	Likely Benign
SGCD		c.*6717G>A		Homozygous	Benign
SGCG		c.*13C>T		Heterozygous	Unknown Significance
SGCG		c.*295T>C		Heterozygous	Likely Benign
SGCG	rs1800354	c.860A>G		Homozygous	Benign
SGSH		c.*315C>T		Homozygous	Benign
SH3TC2		c.3594A>G	p.Pro1198Pro	Heterozygous	Unknown Significance
SH3TC2		c.*2397G>A		Heterozygous	Unknown Significance
SH3TC2		c.*2428C>T		Heterozygous	Unknown Significance
SH3TC2		c.*2900G>T		Heterozygous	Unknown Significance
SH3TC2		c.*3860G>A		Heterozygous	Unknown Significance
SH3TC2		c.*6162C>T		Heterozygous	Unknown Significance
SH3TC2		c.*8756G>T		Heterozygous	Unknown Significance
SH3TC2		c.*10485G>A		Heterozygous	Unknown Significance
SH3TC2		c.*10968G>A		Heterozygous	Unknown Significance
SH3TC2		c.*11120G>A		Heterozygous	Unknown Significance
SH3TC2		c.*11852G>T		Heterozygous	Unknown Significance
SH3TC2		c.*14411G>A		Heterozygous	Unknown Significance
SH3TC2		c.*17317G>A		Heterozygous	Unknown Significance
SH3TC2		c.*19519G>A		Heterozygous	Unknown Significance
SH3TC2		c.*929G>C		Heterozygous	Likely Benign
SH3TC2		c.*3196T>C		Heterozygous	Likely Benign
SH3TC2		c.*7292C>T		Heterozygous	Likely Benign

Illumina Clinical Services Laboratory PG0000644-BLD

MOLECULAR DIAGNOSTICS REPORT

Gene	dbSNP ID	cDNA	AA	Zygosity	Intepretation
SH3TC2		c.*14766T>A		Heterozygous	Likely Benign
SH3TC2		c.*16631G>A		Heterozygous	Likely Benign
SH3TC2	rs1432794	c.1587T>G	p.Arg529Arg	Homozygous	Benign
SH3TC2		c.*3077C>T		Heterozygous	Benign
SH3TC2		c.*3136G>A		Homozygous	Benign
SH3TC2		c.*5490G>A		Heterozygous	Benign
SH3TC2		c.*11390A>G		Heterozygous	Benign
SH3TC2		c.*15823T>C		Homozygous	Benign
SH3TC2		c.*16020A>C		Homozygous	Benign
SH3TC2		c.*16950T>C		Homozygous	Benign
SH3TC2		c.*17703A>G		Homozygous	Benign
SH3TC2		c.*18554G>T		Homozygous	Benign
SH3TC2		c.*19040A>G		Heterozygous	Benign
SH3TC2		c.*19573G>A		Heterozygous	Benign
SH3TC2		c.*19919T>C		Homozygous	Benign
SIX1		c.*334C>G		Homozygous	Benign
SLC16A1	rs1049434	c.1470T>A	p.Asp490Glu	Heterozygous	Benign
SLC16A1		c.*1414C>T		Heterozygous	Benign
SLC17A5		c.*385C>G		Heterozygous	Unknown Significance
SLC17A5		c.*971G>A		Heterozygous	Likely Benign
SLC17A8		c.-218T>C		Heterozygous	Unknown Significance
SLC17A8		c.*39A>C		Homozygous	Likely Benign
SLC22A5		c.*1142C>T		Heterozygous	Unknown Significance
SLC22A5		c.824+13T>C		Heterozygous	Likely Benign
SLC22A5		c.-207C>G		Homozygous	Benign
SLC22A5		c.652+6A>G		Homozygous	Benign
SLC22A5	rs274558	c.807A>G	p.Leu269Leu	Heterozygous	Benign
SLC22A5		c.*843T>C		Homozygous	Benign
SLC22A5		c.*1340A>T		Homozygous	Benign
SLC25A13		c.328+6A>G		Homozygous	Benign
SLC26A4		c.*868G>A		Heterozygous	Likely Benign
SLC3A1	rs3738985	c.114A>C	p.Gly38Gly	Homozygous	Benign
SLC3A1		c.1332+7C>T		Homozygous	Benign
SLC3A1	rs698761	c.1854G>A	p.Met618Ile	Homozygous	Benign
SLC3A1		c.*131T>C		Homozygous	Benign
SLC40A1		c.-98G>C		Homozygous	Unknown Significance
SLC40A1		c.-8C>G		Homozygous	Unknown Significance
SLC7A9	rs35170371	c.399C>T	p.Ser133Ser	Heterozygous	Likely Benign
SLC7A9	rs12150889	c.425T>C	p.Val142Ala	Heterozygous	Likely Benign
SLC7A9	rs11084673	c.507C>T	p.Ser169Ser	Heterozygous	Likely Benign
SLC7A9	rs1007160	c.667C>A	p.Leu223Met	Heterozygous	Likely Benign
SLC7A9	rs1007161	c.687C>T	p.Leu229Leu	Heterozygous	Likely Benign
SLC7A9	rs2287881	c.1143C>T	p.Ala381Ala	Heterozygous	Likely Benign
SLC7A9		c.*79T>C		Heterozygous	Likely Benign

MOLECULAR DIAGNOSTICS REPORT

Gene	dbSNP ID	cDNA	AA	Zygosity	Intepretation
SLC7A9		c.-172T>A		Heterozygous	Benign
SLX4	rs8061528	c.753G>A	p.Ala251Ala	Heterozygous	Likely Benign
SLX4	rs3810812	c.4500T>C	p.Asn1500Asn	Homozygous	Benign
SMPD1	rs1050239	c.1522G>A	p.Gly508Arg	Homozygous	Likely Benign
SMPD1		c.107T>C	p.Val36Ala	Heterozygous	Benign
SNCA		c.*893C>T		Homozygous	Likely Benign
SPTA1		c.1588G>A	p.Glu530Lys	Heterozygous	Unknown Significance
SPTA1		c.1350+14A>T		Homozygous	Likely Benign
SPTA1		c.24+3A>G		Homozygous	Benign
SPTA1	rs435080	c.126C>T	p.Val42Val	Homozygous	Benign
SPTA1		c.813-7A>T		Homozygous	Benign
SPTA1	rs325996	c.942T>A	p.Ala314Ala	Homozygous	Benign
SPTA1	rs2482965	c.3487T>G	p.Ser1163Ala	Homozygous	Benign
SPTB		c.4222G>C	p.Gly1408Arg	Heterozygous	Likely Benign
SPTB		c.4293A>G	p.Arg1431Arg	Heterozygous	Likely Benign
SPTB		c.4476T>C	p.Leu1492Leu	Heterozygous	Likely Benign
SPTB		c.4482G>A	p.Val1494Val	Heterozygous	Likely Benign
SPTB		c.4563+12G>C		Heterozygous	Likely Benign
STK11		c.*616T>C		Homozygous	Benign
SUMF1		c.*471T>G		Homozygous	Unknown Significance
SUMF1		c.*291G>A		Homozygous	Likely Benign
SUMF1		c.*61T>C		Homozygous	Benign
SUMF1		c.*675A>C		Homozygous	Benign
TAT		c.43C>T	p.Pro15Ser	Heterozygous	Unknown Significance
TCAP	rs1053651	c.453A>C	p.Ala151Ala	Homozygous	Benign
TECTA		c.2795T>C	p.Val932Ala	Heterozygous	Unknown Significance
TECTA	rs12275038	c.4098G>A	p.Thr1366Thr	Heterozygous	Likely Benign
TECTA		c.4105+13C>T		Heterozygous	Likely Benign
TECTA	rs612969	c.1111A>G	p.Arg371Gly	Homozygous	Benign
TECTA	rs536069	c.1485A>G	p.Ala495Ala	Homozygous	Benign
TECTA	rs586473	c.2805T>C	p.Tyr935Tyr	Homozygous	Benign
TECTA	rs526433	c.5171G>A	p.Ser1724Asn	Homozygous	Benign
TJP2		c.-185G>A		Homozygous	Unknown Significance
TJP2		c.*183G>A		Homozygous	Likely Benign
TJP2	rs2309428	c.1446C>A	p.Asp482Glu	Homozygous	Benign
TMC1	rs34532421	c.1713C>T	p.Phe571Phe	Heterozygous	Unknown Significance
TMC1		c.-468G>A		Heterozygous	Benign
TMC1		c.-219A>G		Heterozygous	Benign
TMC1	rs2589615	c.45C>T	p.Asp15Asp	Heterozygous	Benign
TMPRSS3	rs2839500	c.757A>G	p.Ile253Val	Heterozygous	Unknown Significance
TMPRSS3		c.*2G>A		Heterozygous	Likely Benign
TMPRSS3		c.*206A>G		Heterozygous	Likely Benign

MOLECULAR DIAGNOSTICS REPORT

Gene	dbSNP ID	cDNA	AA	Zygosity	Intepretation
TMPRSS3		c.447-13A>G		Homozygous	Benign
TMPRSS3	rs2839501	c.453G>A	p.Val151Val	Homozygous	Benign
TNNI3		c.537G>A	p.Glu179Glu	Heterozygous	Unknown Significance
TNNI3		c.25-8T>A		Heterozygous	Likely Benign
TNNI3		c.373-10T>G		Homozygous	Benign
TNNT2		c.207G>A	p.Ser69Ser	Heterozygous	Unknown Significance
TNNT2		c.318C>T	p.Ile106Ile	Heterozygous	Benign
TP53	rs1042522	c.215C>G	p.Pro72Arg	Homozygous	Benign
TPM1		c.*148G>T		Heterozygous	Unknown Significance
TPM1	rs1071646	c.453C>A	p.Ala151Ala	Homozygous	Benign
TPO	rs4927611	c.769G>T	p.Ala257Ser	Homozygous	Unknown Significance
TPO		c.*115G>T		Heterozygous	Unknown Significance
TPO	rs1126797	c.1998C>T	p.Asp666Asp	Heterozygous	Likely Benign
TPO	rs732608	c.2145C>T	p.Pro715Pro	Heterozygous	Likely Benign
TPO		c.*81C>G		Heterozygous	Likely Benign
TPO	rs732609	c.2173A>C	p.Thr725Pro	Heterozygous	Benign
TPO	rs1126799	c.2540T>C	p.Val847Ala	Heterozygous	Benign
TPP1		c.*1588C>T		Homozygous	Likely Benign
TPP1		c.*1628G>A		Homozygous	Benign
TRIM32		c.*129C>T		Homozygous	Benign
TSHR		c.*1152T>A		Heterozygous	Unknown Significance
TSHR		c.*431T>C		Heterozygous	Likely Benign
TSHR		c.*909C>T		Heterozygous	Likely Benign
TSHR	rs1991517	c.2181G>C	p.Glu727Asp	Heterozygous	Benign
TSHR		c.*245C>T		Heterozygous	Benign
TTN		c.1492G>A	p.Val498Ile	Heterozygous	Unknown Significance
TTN		c.7545C>T	p.Tyr2515Tyr	Heterozygous	Unknown Significance
TTN		c.20784C>T	p.Thr6928Thr	Heterozygous	Unknown Significance
TTN	rs2244492	c.28132G>A	p.Gly9378Arg	Heterozygous	Unknown Significance
TTN		c.35254A>G	p.Lys11752Glu	Heterozygous	Unknown Significance
TTN	rs12464787	c.63126C>T	p.Ser21042Ser	Heterozygous	Unknown Significance
TTN	rs56169243	c.64428T>C	p.Gly21476Gly	Heterozygous	Unknown Significance
TTN	rs6715406	c.2244G>A	p.Glu748Glu	Heterozygous	Likely Benign
TTN	rs36051007	c.29555G>A	p.Arg9852His	Heterozygous	Likely Benign
TTN	rs35833641	c.49611T>C	p.His16537His	Heterozygous	Likely Benign
TTN	rs12463674	c.70970T>C	p.Ile23657Thr	Heterozygous	Likely Benign
TTN	rs10497520	c.3601A>G	p.Lys1201Glu	Homozygous	Benign
TTN	rs1552280	c.3884C>T	p.Ser1295Leu	Homozygous	Benign

Illumina Clinical Services Laboratory PG0000644-BLD

MOLECULAR DIAGNOSTICS REPORT

Gene	dbSNP ID	cDNA	AA	Zygosity	Intepretation
TTN		c.4480+6C>T		Homozygous	Benign
TTN	rs2291311	c.9781G>A	p.Val3261Met	Homozygous	Benign
TTN	rs4894043	c.9879A>G	p.Glu3293Glu	Homozygous	Benign
TTN	rs2291310	c.10256G>A	p.Ser3419Asn	Homozygous	Benign
TTN	rs2742348	c.10878C>T	p.Ser3626Ser	Homozygous	Benign
TTN	rs2562831	c.19491G>A	p.Gln6497Gln	Homozygous	Benign
TTN	rs4145333	c.59542G>C	p.Ala19848Pro	Homozygous	Benign
TULP1	rs2064317	c.776T>C	p.Ile259Thr	Heterozygous	Likely Benign
TULP1	rs7764472	c.200C>G	p.Thr67Arg	Homozygous	Benign
TULP1	rs2064318	c.783G>C	p.Lys261Asn	Homozygous	Benign
TULP1		c.*318T>G		Heterozygous	Benign
UBA1		c.811+9C>G		Homozygous	Benign
UGT1A1		c.*211T>C		Homozygous	Benign
UGT1A1		c.*339G>C		Homozygous	Benign
UGT1A1		c.*440G>C		Homozygous	Benign
USH1C	rs10832796	c.1440C>T	p.Val480Val	Homozygous	Likely Benign
USH1C		c.*78T>C		Homozygous	Likely Benign
USH1C		c.*218C>T		Homozygous	Likely Benign
USH1C		c.*243A>G		Homozygous	Likely Benign
USH1C	rs1064074	c.1557G>C	p.Glu519Asp	Homozygous	Benign
USH2A	rs10779261	c.373G>A		Homozygous	Benign
USH2A	rs4253963	c.504A>G		Homozygous	Benign
USH2A	rs1805049	c.4457G>A		Homozygous	Benign
VAPB		c.*937G>C		Heterozygous	Unknown Significance
VAPB		c.*1282A>G		Heterozygous	Unknown Significance
VAPB		c.*5250T>C		Heterozygous	Likely Benign
VAPB		c.*5298C>T		Heterozygous	Likely Benign
VCL	rs767809	c.2388G>A	p.Pro796Pro	Homozygous	Benign
VCL	rs2131956	c.2814C>G	p.Gly938Gly	Homozygous	Benign
VCL		c.*1584T>A		Homozygous	Benign
WFS1		c.1023C>T	p.Phe341Phe	Heterozygous	Unknown Significance
WFS1		c.1725C>T	p.Ala575Ala	Heterozygous	Unknown Significance
WFS1		c.2322G>A	p.Lys774Lys	Heterozygous	Unknown Significance
WFS1		c.*90G>A		Heterozygous	Unknown Significance
WFS1		c.*253G>A		Heterozygous	Unknown Significance
WFS1	rs1801212	c.997G>A	p.Val333Ile	Heterozygous	Likely Benign
WFS1	rs1801213	c.684C>G	p.Arg228Arg	Heterozygous	Benign
WFS1		c.2565A>G	p.Ser855Ser	Heterozygous	Benign
WFS1		c.*47T>C		Heterozygous	Benign
WFS1		c.*91C>T		Heterozygous	Benign
