# Supplementary material for: Integrating precision medicine in the study and clinical treatment of a severely mentally ill person
Source: PeerJ. 2013 Oct 3;1:e177. doi: 10.7717/peerj.177 (PMC3792182; doi:10.7717/peerj.177)
Supplement: File S11 [file peerj-01-177-s016.docx]

**Supplementary Methods**

***Description of the surgical procedure for DBS implantation***

All surgery was conducted by a neurosurgeon.

1. Placement of stereotactic head frame.

After administration of prophylactic antibiotics, M.A. was transported to the MRI suite. In the treatment room, M.A. was seated in a wheelchair and the stereotactic head frame was assembled and placed on his head.

2. Alignment of M.A. in stereotactic head frame for computerized tomography and magnetic resonance imaging.

The head of M.A. was partially shaved. Pin sites were prepped sterilely and then bolts were tightened down. Once the CRW frame was in place, M.A. was brought to the CT scanner and the Sugita head frame was matched to the CT table and a CT was placed. A CT scan was obtained in a parallel manner. 2.5 mm axial cuts were obtained (Fig. 1: **A**). All findings were well defined in the same cut, and the anterior and posterior commissure lines were identifiable in the same cut. With the CRW head frame and undercage in place, M.A. was then brought to the MRI suite and placed supine on the Gantry table. M.A.’s head was then aligned orthogonally in a head coil and wedged in position to reduce motion artifact. Multiple images and series per the DBS protocol were then obtained. The red nuclei, fornices, AC and PC lines were all identifiable and symmetrically aligned. Once images were obtained, M.A. was then transferred back to the holding area.

4. Evaluation of magnetic resonance imaging and computerized tomography images and computer assisted image fusion and targeting.

Images were then transferred to a computer work station where they were reformatted and bundled. The CT sequences were then fused to the corresponding MRI sequences and then used to select anatomical landmarks. The stereotactic coordinates for the visualized target structures were calculated. Coordinates were also compared to the Shetland brand stereotactic Allis coordinates as well. Once target points had been determined and appropriate entry point in the skull chosen, the connection from entry point to target point was made. Once optimal trajectory was obtained, images were then saved.

6. Left and right frontal burr hole for placement of multiple microelectrode recording arrays.

With the stereotactic head frame in place, M.A. was brought to the operating room and placed in the supine position. A Foley was placed and prophylactic IV antibiotics were then administered. The head frame was then attached to the bed to make the head stable. The head was then prepped and draped with Betadine paint and scrub, and then the fluoroscopic machine was draped in place for later use. Local anesthetic was then injected into the area of the planned incision on both the right and left side, just anterior to the coronal suture. The stereotactic arch was assembled and then adjusted to the zero point on the back table. It was then placed on M.A.'s head frame and tightened down. The neurosurgeon then used it to adjust to proper stereotactic coordinates and to plan the area of incision, first on the right side. A #10 blade scalpel was then used to incise the skin over the area of planned incision and the burr hole location was again confirmed using the stereotactic head frame and arc. The high-speed air drill was then used to create a burr hole which was subsequently widened slightly using Kerrison punches as necessary. The dura was then opened using Bovie electrocautery, with burning and sealing of any small cortical veins. After the burr hole insert was screwed into place, the stereotactic arc was then placed back on to its appropriate settings and the alpha-omega microdriver base was then attached to the arc and then 3 brain cannulas were slowly advanced 25 mm proximal to the predetermined target with the posterior cannulas offset by 2 mm from the center cannula in a Y-plane. Stylettes were removed and the 3 recording electrodes were loaded in the cannulas. The neurosurgeon then slowly advanced the electrodes toward the intended target while recording. The procedure was repeated on the left side in an identical manner

8. Left and right-sided microelectrode stimulation.

The neurosurgeon performed a period of testing while M.A. was awake. Neuropsychiatric testing was performed while stimulating the nucleus accumbens and M.A. had an adequate response. The neurosurgeon thus chose a single target and then placed a final electrode in that location and removed the other 3 microelectrodes. The neurosurgeon left that final electrode in place and removed the microdriver and then attached the DBS stimulating electrode into the navigating insert. Fluoroscopy was used to confirm location and to make sure that the tip could not move during the remainder of the manipulation. The neurosurgeon then repeated the procedure on the left side in an identical manner.

10. Left and right deep brain stimulator lead placement to nucleus accumbens.

A small plastic bullet was placed on the end of the electrode itself and then it was tunneled into the scalp just over the left ear. The neurosurgeon then repeated the procedure on the left side in an identical manner.

12. Confirmatory fluoroscopy.

13. Confirmatory head computerized tomography.

14. Placement of deep brain stimulator generator in left chest pocket.

Once both electrodes were tunneled just above the left ear in the scalp subcutaneously, we then proceeded to close the cranial wounds and remove the stereotactic ring from the head. The arc was removed and the stereotactic head frame left in place. The neurosurgeon then closed the cranial wounds with 2-0 Vicryl interrupted fashion, followed by closure of the skin with a running interlocking nylon. All sponge and needle counts were correct at the end of the portion of this procedure. The neurosurgeon then broke down and prepped and draped M.A.'s left side including chest, neck, and including the area where the DBS electrodes had been tunneled just behind the ear. The stereotactic head frame was removed. M.A. then was prepped and draped in the usual sterile fashion. The neurosurgeon used a 10 blade scalpel to open just above the area were the DBS electrodes had been tunneled. Bovie electrocautery was cauterized down to reveal the bullets that were on the end of the electrodes and those bullets were removed and brought out through that small incision. A tunneling device was then used to tunnel from the scalp down the subcutaneous tissues of the neck, above the collar bone, and then a #10 blade scalpel was used to create a pocket in the chest for the generator to sit. The neurosurgeon then passed the electrode extension up the tunneler and then removed the tunneler. This left an extension lead tunneled from the chest wound up to the posterior auricular wound. The neurosurgeon then copiously irrigated all the wounds with antibiotic containing solution, attached the generator in the usual manner and then tacked 1 small 2-0 silk stitch into the clavicular fascia and then attached that to the generator to make sure that the generator would not migrate. The generator was placed in the pocket and then a small stitch was tightened and the neurosurgeon then placed vancomycin powder into that wound and closed it with 2-0 Vicryl in interrupted fashion to close the deep tissue followed by closure of the skin with 2-0 nylon in a running fashion. The neurosurgeon then attached the extension leads to the DBS electrodes, making sure to keep track of which was left and which was right. Once attached, the remaining wound was closed with 2-0 Vicryl in the fascia followed by closure with 2-0 nylon in running interlocking fashion. All sponge and needle counts were correct at the end of the case.

***CLIA whole genome sequencing***

The sequence was generated from extracted DNA, sequenced using the Illumina HiSeq 2000 sequencer. Briefly, DNA was fragmented, and the fragments were attached to the surface of a glass microscope slide. The fragments were then sequenced using fluorescently labeled nucleotides, which were excited by a laser and imaged using digital equipment. These fragments were then assessed for quality using a variety of metrics to ensure that only robust sequences were analyzed. Fragments were aligned to the NCBI reference sequence. Fragments that aligned to more than one region of the reference genome were excluded from the report. Additionally, fragments were excluded from the analysis on the basis of quality and alignment scores. Each nucleotide site reported was sequenced an average of 30 times, so there was on average 30-fold redundancy for each base pair reported. Additionally, no positions were called when the genotype quality score was less than 30 or depth was less than 6. Only single nucleotide variants are called and validated in the Illumina Clinical Services Laboratory.

***Genotyping array data***

Genotyping array data was generated in parallel of the CLIA whole genome sequencing, using the Illumina HumanOmni2.5-8 bead chip. The encrypted hard drive contains several files, including a “genotyping folder” within which there is a genotyping report (see Supplemental File S1) in a text-based tab-delimited format and including a header with a minimum of the following columns:

*SNP Name:* The snp identifier. An rsID for dbSNP content, Illumina labels otherwise.

*GC Score:* This score is a quality metric that generally indicates reliability of the genotypes called. The GenCall score is a value with a maximum of 1 assigned to every genotype called. GenCall scores are calculated using information from the clustering of the samples. Each SNP is evaluated based on the angle of the clusters, dispersion of the clusters, overlap between clusters, and intensity. Genotypes with lower GenCall scores are located furthest from the center of a cluster and have a lower reliability.

*Allele A* – Forward: A allele call relative to the Forward sequence as defined by the SNP source database (dbSNP, 1000Genomes, etc.)

*Allele B* – Forward: B allele call relative to the Forward sequence as defined by the SNP source database (dbSNP, 1000Genomes, etc.)

*Allele A* – Design: A allele call relative to the probe used.

*Allele B* – Design: B allele call relative to the probe used.

***Omicia Opal software***

Omicia Opal is implemented in software-as-a-service model. All user interactions take place through web browsers using a secure https protocol. Upon upload of the variant files (vcf or gvf format), the genomes are processed by Omicia’s Opal Annotation Pipeline and the Omicia’s VAAST analysis tool. Computationally annotated variants are loaded into Opal’s relational database for further variant mining by users through the Opal web interface. The system is currently accessible through a 128 bit encrypted connection and HIPAAcompliant. Each user is given access to only their own data, or data that the user elects to share with others by explicitly giving them permission through the user interface.

***Omicia’s Opal Annotation Pipeline***

Opal’s pipeline annotates all variants in the genome variant file. Annotations include synonymous, non-synonymous, slice site, and stop gain/loss SNPs as well as small indels (25bp or less that may induce frame-shifts.) To be retained for use in the Variant Mining interface and other applications, a variant must satisfy either of the following criteria:

Overlap a protein-coding region of a known gene (ENSEMBL database v62)

Be annotated in any of the following sources containing known pathogenic variants:

OMIM: Online Mendelian Inheritance in Man (http://www.ncbi.nlm.nih.gov/omim)

HGMD: Human Gene Mutation Database (version 2010.2, http://www.hgmd.org/)

PharmGKB: Pharmacogenomics Knowledge Base (http://www.pharmgkb.org/)

LSDB: Locus-specific mutation databases (from PhenCode, http://phencode.bx.psu.edu/otherLinks.html)

GWAS: NHGRI Catalog of Published Genome-Wide Association Studies (http://www.genome.gov/26525384)

Data Source Versions

dbSNP - release 135

ENSEMBL - version 62 ("homo_sapiens_core_62_37g")

HGMD - version 7.1

DrugBank (downloaded 11/2011)

OMIM (downloaded 11/2010)

PharmGKB (downloaded 9/2010)

1000 Genomes - phase I release (downloaded 12/2011)

Variants are defined as a sequence change from either the GRCh37/hg19 or NCBI36/hg18 genomic reference sequence. The variant files can contain whole genome, exome or targeted next-generation sequencing data from various platforms. Acceptable formats are VCF, Genome Variant Format (GVF), and the Complete Genomics’ masterVar format. Upon upload, the Omicia Opal system processes each variant list through a series of annotation programs called the Omicia Opal Annotation Pipeline. The Annotation Pipeline runs on Omicia’s servers and annotates variants using the following multi-step workflow. The version of the genomic reference sequence used in variant file generation is verified by comparison to known polymorphisms in the NCBI dbSNP 135 database[[1](#_ENREF_1)]. If more than 60% of the SNVs in an exome or genome file have positions on a particular version of the genomic reference sequence identical to known dbSNP entries, then that version is assigned to the variant file. For smaller numbers of variants, as seen in targeted-sequence projects, the pipeline relies strictly on user-supplied genomic reference annotation. The pipeline then gathers a number of frequently-used summary statistics about the files, including the number of variants, median Phred-like quality[[2](#_ENREF_2)] of variants, median reads per variant, and transition/transversion (ti/tv) ratio. Genomes and exomes for which these measures fall more than two standard deviations from the median value in previously observed genomes or exomes are flagged as being problematic. A score is derived from these parameters, and noted as the Omicia Genome Clinical Grade (for details see app.omicia.com). The pipeline then classifies the function of each variant. Each variant is classified according to the location within and effect on the protein. Variants in protein coding regions are classified as synonymous, non-synonymous, stop-gained, stop-lost, frameshift insertion/deletion, non-frameshift insertion/deletion, and splice-site variants. Variants outside of protein coding regions are classified as either 3’ untranslated region (UTR), 5’UTR, intronic, intergenic, splice-site or splice region variants. The Omicia system uses a combination of the Ensembl Database release 62[[4](#_ENREF_4)] and RefSeq[[5](#_ENREF_5), [6](#_ENREF_6)] databases as a basis for these classifications and presents the variant and protein change in the Human Genome Variation Society (HGVS)[[7](#_ENREF_7)] nomenclature. The annotated gene names use the official HUGO Gene Nomenclature Committee (HGNC) gene symbols[[8](#_ENREF_8)]. If the variant is present in the NCBI dbSNP 135 database, then the variant is annotated with the dbSNP identifier (rs number). Variants found in the Online Mendelian Inheritance in Man (OMIM)[[9](#_ENREF_9)] database of disease-causing mutations have a specific OMIM hyperlink to the variant. OMIM variants are typically described in coordinates relative to protein sequences as described at the time of their publication, and, over time, these variant coordinates can become invalid as the reference genome is updated. The Omicia pipeline uses a proprietary variant alignment algorithm in order to annotate the variants’ position on the hg19 version of the reference genome. Variant annotations (and if available, hyperlinks) are also given for the following databases: the Human Genome Mutation Database, version 7.2 (HGMD)[[10](#_ENREF_10)], Phencode collection of locus-specific databases[[11](#_ENREF_11)]**,** the National Human Genome Research Institute (NHGRI) Catalog of Published Genome-Wide Association Studies (GWAS), and the Pharmacogenetics Knowledge Base (PharmGKB)[[12](#_ENREF_12)]. In addition, variants are annotated with the allele and genotype frequency information from the 1000 Genomes Project[[13](#_ENREF_13)]. This information can be used to distinguish common polymorphisms from rare, possibly disease-causing mutations.

Next, scores predicting pathogenicity are generated for each protein-coding variant using the following programs: SIFT[[14](#_ENREF_14)], PolyPhen 2[[15](#_ENREF_15)], MutationTaster[[16](#_ENREF_16)], and PhyloP[[17](#_ENREF_17)]. The Sorting Intolerant from Tolerant (SIFT) scores are a prediction of the tolerance for certain amino acid changes within the protein and are based on evolutionary conservation at that protein position. SIFT p-values below 0.05 indicate the change is likely deleterious. PhyloP assesses the evolutionary conservation of each position. The PhyloP score is the –log (p-value) under a null hypothesis of neutral evolution, and a negative sign indicates faster-than expected evolution, while positive values imply conservation. PolyPhen 2 is a tool that predicts possible impact of an amino acid substitution on the structure and function of a human protein using a number of structural, physical, and comparative considerations. This algorithm produces one of three calls for non-synonymous variants: benign, possibly damaging, and probably damaging. MutationTaster predicts whether protein-coding variants are disease-causing or benign and assigns a P-value to these predictions. Each variant is further assessed for pathogenicity using a proprietary algorithm, the Omicia Variant Score, a random-forest classifier [[18](#_ENREF_18)] that takes as input the Polyphen, SIFT, MutationTaster and PhyloP score, and comes up with an integrative score between 0-1, called the Omicia Score. The classifier was trained using disease-causing “DM” mutations from the HGMD database and a similarly-sized set of SNPs from dbSNP132 with a minor allele frequency above 5% assumed benign. A randomly chosen subset of 10,000 variants was left out of the combined training set and used in validation. The underlying classifier assigns each variant an either “benign” or “pathogenic” classification and a confidence value between 0 and 1 for that classification. To enable simple filtering strategies, these classifications are scaled to a single-valued 0-1 scale with 1 corresponding to variants that the Assessor has highest confidence in being pathogenic and 0 corresponding to variants the Assessor determines are most likely to be benign. A score of 0.85 or higher generates a 1% false positive prediction rate within our testing set. A variant score higher than 0.85 is considered to be likely pathogenic and a score between 0.5 and 0.85 is considered potentially pathogenic. Finally, the output of the Annotation Pipeline is loaded into a data repository utilized in the Variant Miner view. For further details on the method and the program see [www.omicia.com](http://www.omicia.com).

**Pharmacogenomic Analysis from G.H. – AssureRx Health**

***Advanced Pharmacogenomic Knowledge Base - GenomePharm (See Figure 1 below):***

This biomedical informatics platform is still under development, but consists of the following components:

1. Query-based, faceted search capabilities.
2. Service Oriented Architecture (SOA).
3. Access to private / proprietary data contained in primary data sources from pharmaceutical & biotechnology companies, academia, & publishers such as Thomson-Reuters, through a pre-competitive data-sharing community called ‘tranSMART’ (see [www.transmartfoundation.org](http://www.transmartfoundation.org) ).
4. Natural language processing of unstructured text from both longitudinal de-identified EHRs and from clinical trial data from [www.clinicaltrials.gov](http://www.clinicaltrials.gov) .
5. Access to a database of 17,131 whole genome data, generated under federal contract to the U.S. Army. Approval established through the U.S. Army’s Institutional Review Board.
6. Access to drug databases from First Databank, Cerner, and Thomson Reuter under license.
7. Applications layer, including open source software such as R, KEGG and Biomart, as well as donated software APIs including Spotfire® (Perkin Elmer & TIBCO software).
8. Access to public resources in cloud, including FAERS and iAEC, published literature, NCBI resources, *etcetera.*
9. Standardized heterogeneous database service, based on OWL-S (ontology web language service).


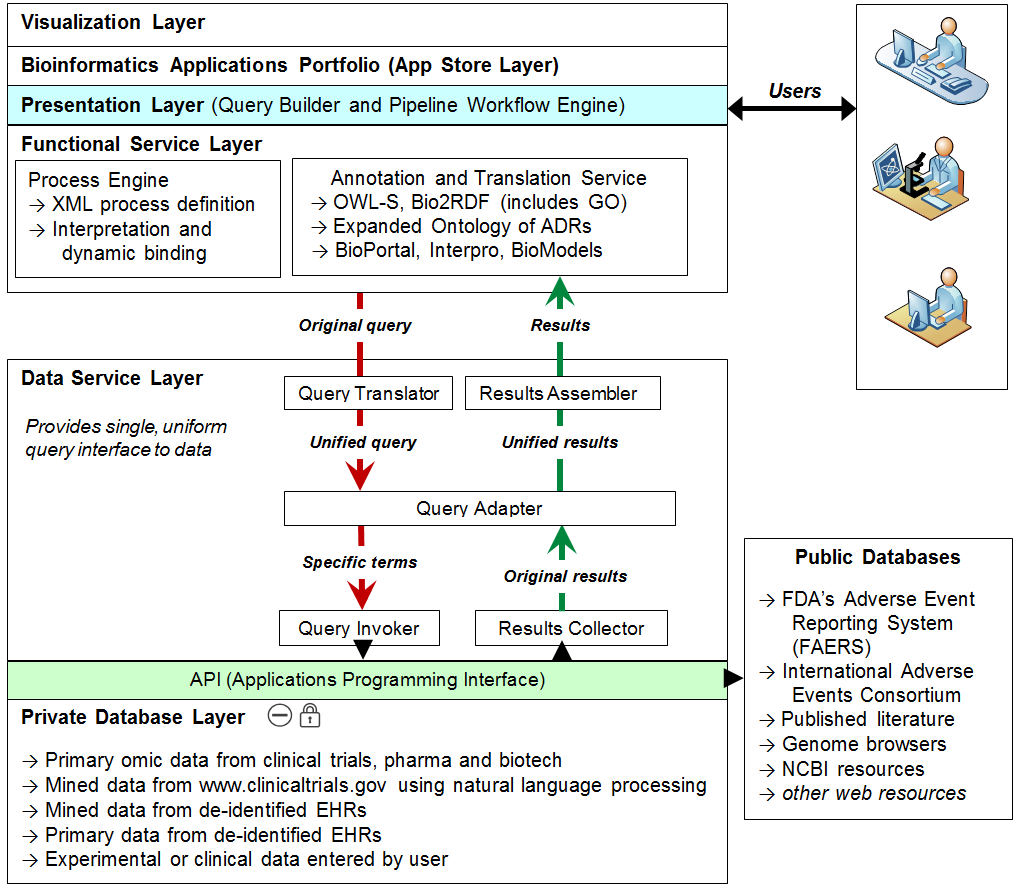


Figure 1. Architecture of the advanced pharmacogenomic knowledge base- GenomePharm.

**Supplementary Pharmacogenomic Analysis by G.H.**

All single nucleotide variants (SNPs), copy number variants (CNVs), indels and other variants identified as important and provided by Omicia’s Opal Annotation Pipeline were investigated further using GenomePharm and manual review of the published literature and clinical trial data. These were compared to genes listed in Pharma DMET, Pharma ADME, and the ADME Pharma consortium. Comparisons were made of known gene-variant-drug-disease interactions as identified in GenomePharm and stored in a temporary NoQL database. Data were exported to an Excel spreadsheet for Supplementary Material. Judgments were made by G.H., in which a given pharmacogenomic variant had to have been replicated >6 times in adequately powered randomized controlled trials using individuals of European American ancestry with determination of the following:

1. **1 − β**: One minus the Type II error rate;
2. ***R:*** Ratio of the number of “true relationships” to “no relationships” among those tested in the field; *and*
3. ***PPV***: Positive predictive value.

Data were obtained from multiple data sources, including clinical trials and published literature.

**Detailed Information written by and provided by M.A.:**

**“Treatise on my extreme OCD with psychotic features”**

The majority was written by M.A. on August 30, 2012.

This is an attempt to capture the sense of psychotic delusions, brought on by the following disorders: delusional OCD with poor insight, OCD with psychotic features, or OCD with co-morbid schizoaffective disorder.  These have all been diagnoses I have received in the past.  The diagnosis doesn't really matter. In this column, I wanted to capture the loneliness, despair, and anguish that comes with the struggle to fight suicidal ideations that accompanies extreme mental illness.

I erased most of my scary poems written while severely mentally ill, but I kept a few. I received experimental brain surgery and am no longer suicidal.  I received Deep Brain Stimulation last year, but I felt that this sense of loss, and pain that I wrote about was important to remember.  However, at the time, it seemed like I advocated suicide for everyone, so I decided to add some encouragement to others suffering from severe to extreme mental illness.

I never could have predicted that during my worst times, that some day I would receive an amelioration of symptoms. I felt like my mental illness was incurable because I had tried at least 30 medicines, tried every type of counseling, tried every quack treatment advertised in nonprofessional journals and enrolled in every experiment advertised in professional journals, and my symptoms kept getting worse. However, brain surgery was something I hadn't tried, and I wish I had gotten the surgery 20 years ago, and saved myself needless suffering. However, I don't want to imply that I was cured, I merely suffer less in agony.  And my depression and psychosis is in complete remission when the battery is on and when I receive my biweekly shots of long lasting antipsychotics.

One of the subtypes of OCD that I suffered from was scrupulosity. The gist of scrupulosity obsessions with OCD is to obsess about the worst crimes imaginable and fear losing control and one day committing them.  I learned to manage the self-loathing and self hatred that comes with scrupulosity. I would perform compulsions, which are irrational actions or rituals performed repetitively to manage anxiety. My compulsions were to fantasize about killing myself or mutilating myself in the most painful ways I could imagine.  I did this because I considered myself a murderer who had simply been too lazy and cowardly and thus not gotten around to my first crime.  However, there are a lot of crimes much worse than murder and I have committed every one of these crimes thousands of times in my mind and then punished myself compulsively for the obsessions.

However, during my time in the military, after some traumatic events and including some head traume, I became psychotic.  For me psychosis entailed extreme confusion, memory loss, delusions, paranoia, and other symptoms.  Psychosis is different for everyone, but for most people psychosis does not mean that they are a walking time bomb of antisocial behavior.  For me, I was way too confused to commit violence, or any other crime.  Statistically people with schizophrenia are more likely to have violence committed against them than to commit violence against the world's healthy population.

When I was psychotic and delusional I would remember all the horrible crimes I had previously obsessed about, and then figure I was actually guilty of committing these crimes in real life.  However, even though I was delusional, I was still always confused, because I couldn't remember why I had done these horrible deeds or where or when. The only information I had was about how I committed these crimes and that was vividly engraved in my memory. Because of my fear of being arrested and tortured for my sins, after becoming delusional I would usually wander the streets or lock myself up in my room thinking I was the worst murderer, rapist, torturer, etc. person in the world.

If I wasn't psychotic I would have killed myself over the shame and guilt, but at those times, I was usually too sick to act on suicidal ideations.  However, when police or family members would take me into the VA for observation because I was muttering incomprehensively to myself or living in complete filth, the doctors would put me on antipsychotics and I would come back to reality.  However, it was an even more hellish reality than before my psychotic breaks.  Because after each psychotic episode, I was left with disturbed memories of committing the worst atrocities imaginable to family members and old friends long since abandoned .

When you are worried that you are the most despicable monster the world has ever seen, you cut yourself off from dating, friends, coworkers, and even family. However, these memories were obviously false.  However, my parents and brothers and sisters got tired of me asking them did I kill/rape/torture you last month. My psychiatrists looked at me strangely when I told them I had killed my family members when they knew those family members because they met them when I was usually committed and when discharged.  I learned I could not share these traumatic memories with anyone because every time I did, I was hospitalized. I soon realized I was the only one who believed these delusional memories. OCD however, is a disease of irrational fears and even my occasional insight into having a disease was no comfort from the shame and guilt for being the worst monster the world has ever known. Besides the fact that these distorted memories haunted me as flashbacks and nightmares at all times.

I learned to find order within my confused memories.  Memories that had no end or beginning were suspect as delusional.  If I killed someone but did not remember hiding the body and evidence, did not remember thinking up elaborate alibis, did not remember feeling guilt or shame, or anger, then the memory was suspect.  All these responses to murder occur to people who actually commit murder.  I only had vivid memories of the actual present commission of the murder deed and nothing else.  Besides the fact that all the people I murdered are still alive and still love me.  Of course, I had irrational obsessions, that they were all secretly dead who I killed in my delusions, but I didn't know why they all seemed to have forgiven me so carelessly for my heinous acts of murdering them.

However, as I said earlier, the nature of OCD is to obsess about irrational things that cause you pain and disturb you, and then act on even stranger less rational compulsions to deal with the pain.  I spent literally days (up to 120 hours without sleep) awake obsessing about the perceived crimes and then punishing myself for these crimes I had committed, even though I was beginning to realize that these memories were false.

However, these memories were just as disturbing as any real memory.  I still can not contrast these memories from real memories other than the fact that like a nightmare the false memories did not have beginning or end, they only exist in the present event without precursors or after effects.  One of my psychiatrists felt I should be diagnosed with PTSD if not for the obvious OCD diagnosis. They are related anxiety disorders. This is because I suffered traumatic flashbacks from both traumas received in the military and for the repeated trauma of reliving all my delusional memories, which included the worst traumas imaginable committed to those I loved most, in obsessive detail.  Even while asleep I could find no relief from these traumatic obsessions.

Of course this is another reason why I would stay awake for 3 to 5 days, besides the inability to stop obsessing over my atrocities, as a way to keep me from violently reliving the nightmares while I slept.  If I stayed awake long enough, I slept the sleep of the damned without being able to remember nightmares when I awoke.

Post surgery I still have moderate to severe OCD. My YBOCS score is anywhere from 28-32. YBOCS stands for Yale Brown Obsessive Compulsive Scale. Pre-surgery, my scores were 38-40. This is meaningless minutiae to anyone who hasn't taken the test. But the maximum score is 40 with 16 hours in row spent daily obsessing and compulsions. Of course the fact that I would stay awake for 72 to 120 hours in a row stuck on obsessions and compulsions doesn't change the score any.

However, post surgery most of my delusional obsessions, relating to scrupulosity and the psychotic delusional episodes are in remission.  My extreme depression is also in remission.  I still have a hard time functioning like a healthy individual, but I am improving.  I have gone back to work part time.  My current poetry I write is much healthier, less angry, not suicidal, and not disturbing.  I now feel tired and hungry as appropriate as well as sated and well rested as appropriate. I also feel a full ranges of emotions, rather than numbness.

I want everyone who reads this to understand that I was one of the sickest of the sick in terms of mental illness, and I was sick for over 15 years never able to predict if I would be able to cope for longer than 2 days without resorting to suicide.  However, unlike my predictions, medical advances caught up with me and some of my mental illness is currently in remission post experimental brain surgery.  I say remission and not cure, because I go right back to where I was when I do not charge my pacemaker in my brain each night or if I stopped taking my antipsychotic shot.

I still believe that for some people suicide is a dignified way to end terminal suffering.  However, for many people with suicidal thoughts and for most people with mental illness, the suicidal ideation makes one unfit to make reasoned judgments concerning the nature and length of the suffering.  I thought my suffering was terminal and I now realize that though it indeed felt terminal and seemed that long, in reality I suffered for but a short time, and because of medical advances my torture proved curable to some extent.

We don't know the future and can't predict where medicine will bring us in the future.  I read that everyone who suffers from paralysis feels deeply suicidal at first, but almost all people who become paralyzed come to find joy in their new lives. So I encourage all who seek death to postpone the inevitable if only for two days, and then make those two days stretch out much longer with thousands of death procrastinations, until medicine can bring relief. Even though, I still suffer horribly, there are moments when I truly smile for real and that makes the rest of my life worth bearing.

**Supplementary References**

1. Jones MK, Wootton BM, Vaccaro LD: **The efficacy of exposure and response prevention for geriatric obsessive compulsive disorder: a clinical case illustration.** *Case reports in psychiatry* 2012, **2012:**394603.

2. Ewing B, Green P: **Base-calling of automated sequencer traces using phred. II. Error probabilities.** *Genome Res* 1998, **8:**186-194.

3. Wang K, Li M, Hakonarson H: **ANNOVAR: functional annotation of genetic variants from high-throughput sequencing data.** *Nucleic Acids Res* 2010, **38:**e164.

4. Schwarz JM, Rodelsperger C, Schuelke M, Seelow D: **MutationTaster evaluates disease-causing potential of sequence alterations.** *Nat Methods* 2010, **7:**575-576.

5. National Library of Medicine (US) NCfBI: **The NCBI handbook [Internet], Chapter 18, The Reference Sequence (RefSeq) Project. .** 2002.

6. Pruitt KD, Tatusova T, Klimke W, Maglott DR: **NCBI Reference Sequences: current status, policy and new initiatives.** *Nucleic Acids Res* 2009, **37:**D32-36.

7. den Dunnen JT, Antonarakis SE: **Mutation nomenclature extensions and suggestions to describe complex mutations: a discussion.** *Hum Mutat* 2000, **15:**7-12.

8. Seal RL, Gordon SM, Lush MJ, Wright MW, Bruford EA: **genenames.org: the HGNC resources in 2011.** *Nucleic Acids Res* 2010, **39:**D514-519.

9. Phillips KA, Gunderson CG, Mallya G, McElroy SL, Carter W: **A comparison study of body dysmorphic disorder and obsessive-compulsive disorder.** *The Journal of clinical psychiatry* 1998, **59:**568-575.

10. Hung TC, Tang HS, Chiu CH, Chen YY, Chou KR, Chiou HC, Chang HJ: **Anxiety, depressive symptom and suicidal ideation of outpatients with obsessive compulsive disorders in Taiwan.** *Journal of clinical nursing* 2010, **19:**3092-3101.

11. Coonrod EM, Durtschi JD, Margraf RL, Voelkerding KV: **Developing Genome and Exome Sequencing for Candidate Gene Identification in Inherited Disorders.** *Arch Pathol Lab Med* 2012.

12. Alonso P, Segalas C, Real E, Pertusa A, Labad J, Jimenez-Murcia S, Jaurrieta N, Bueno B, Vallejo J, Menchon JM: **Suicide in patients treated for obsessive-compulsive disorder: a prospective follow-up study.** *Journal of affective disorders* 2010, **124:**300-308.

13. Li H, Homer N: **A survey of sequence alignment algorithms for next-generation sequencing.** *Brief Bioinform* 2010, **11:**473-483.

14. Hu H, Wrogemann K, Kalscheuer V, Tzschach A, Richard H, Haas SA, Menzel C, Bienek M, Froyen G, Raynaud M, Van Bokhoven H, Chelly J, Ropers H, Chen W: **Mutation screening in 86 known X-linked mental retardation genes by droplet-based multiplex PCR and massive parallel sequencing.** *Hugo J* 2009, **3:**41-49.

15. Ghosh S, Krux F, Binder V, Gombert M, Niehues T, Feyen O, Laws HJ, Borkhardt A: **Array-based sequence capture and next-generation sequencing for the identification of primary immunodeficiencies.** *Scand J Immunol* 2012, **75:**350-354.

16. Bejar R, Stevenson K, Abdel-Wahab O, Galili N, Nilsson B, Garcia-Manero G, Kantarjian H, Raza A, Levine RL, Neuberg D, Ebert BL: **Clinical effect of point mutations in myelodysplastic syndromes.** *N Engl J Med* 2011, **364:**2496-2506.

17. Pollard KS, Hubisz MJ, Rosenbloom KR, Siepel A: **Detection of nonneutral substitution rates on mammalian phylogenies.** *Genome Res* 2010, **20:**110-121.

18. Breiman L: **Random Forests.** *Machine Learning* 2001, **45:**5-23.
